# Endoplasmic reticulum-localized circular RNA FAM13B restrains nasopharyngeal carcinoma lymphatic metastasis through downregulating XBP1

**DOI:** 10.1186/s13046-025-03468-7

**Published:** 2025-07-31

**Authors:** Guo-Dong Jia, Si-Yi Xie, Xiao-Yun Li, Liang-Ji Li, Jing Jin, Li-Ting Liu, Xue-Song Sun, Sai-Lan Liu, Qiu-Yan Chen, Lin-Quan Tang, Li Yuan, Hai-Qiang Mai

**Affiliations:** Department of Nasopharyngeal Carcinoma, Sun Yat-sen University Cancer Centre, State Key Laboratory of Oncology in South China, Collaborative Innovation Centre for Cancer Medicine, Guangdong Key Laboratory of Nasopharyngeal Carcinoma Diagnosis and Therapy, 651 Dongfeng Road East, Guangzhou, 510060 People’s Republic of China

**Keywords:** Nasopharyngeal carcinoma, Lymphatic metastasis, Endoplasmic reticulum stress, CircRNA

## Abstract

**Background:**

Nasopharyngeal carcinoma (NPC) is often diagnosed at an advanced stage due to its hidden location, with 70–80% of patients presenting with cervical lymph node metastasis. This high metastasis rate is a major cause of treatment failure and mortality. Non-coding RNAs, particularly circRNAs, have emerged as key players in tumor development, but their roles in NPC lymph node metastasis and angiogenesis remain unclear. This study aimed to identify key circRNAs involved in NPC lymph node metastasis and elucidate their mechanisms of action.

**Methods:**

We identified circFAM13B, a differentially expressed circRNA, using transcriptome sequencing of nasopharyngeal tissues from patients with and without lymph node metastasis. Stable cell lines with circFAM13B overexpression and knockdown were constructed. Functional experiments, including cell invasion, migration, and metastasis assays, were performed in vitro and in vivo. Mechanistic studies involved RNA sequencing, RNA pull-down, and RNA immunoprecipitation assays to explore interacting proteins and signaling pathways.

**Results:**

CircFAM13B was downregulated in metastatic tissues and localized in the endoplasmic reticulum (ER). It acted as a tumor suppressor by binding to RBM3 and promoting degradation of uXBP1 mRNA, a key ER stress molecule. This interaction downregulated sXBP1 and CST6, inhibiting lymphangiogenesis and metastasis. Reduced CST6 expression was associated with poor prognosis in NPC.

**Conclusions:**

Our study reveals that circFAM13B inhibits ER stress-related pathways in NPC, highlighting its potential as a therapeutic target for lymph node metastasis.

**Supplementary Information:**

The online version contains supplementary material available at 10.1186/s13046-025-03468-7.

## Significance

Our study identifies a novel mechanism by which circFAM13B, a circular RNA localized in the endoplasmic reticulum (ER), regulates lymph node metastasis in nasopharyngeal carcinoma (NPC). This unique subcellular localization of circFAM13B is a key innovation, as it highlights the involvement of ER stress pathways in NPC progression. By binding to RBM3 and promoting the degradation of uXBP1 mRNA, circFAM13B downregulates sXBP1 and CST6, thereby inhibiting lymphangiogenesis and metastasis. This work not only underscores the importance of circRNA subcellular localization in cancer biology but also provides a potential therapeutic target for NPC, a disease with high metastatic potential and limited treatment options.

**Supplementary Information:**

The online version contains supplementary material available at 10.1186/s13046-025-03468-7.

## Graphical Abstract

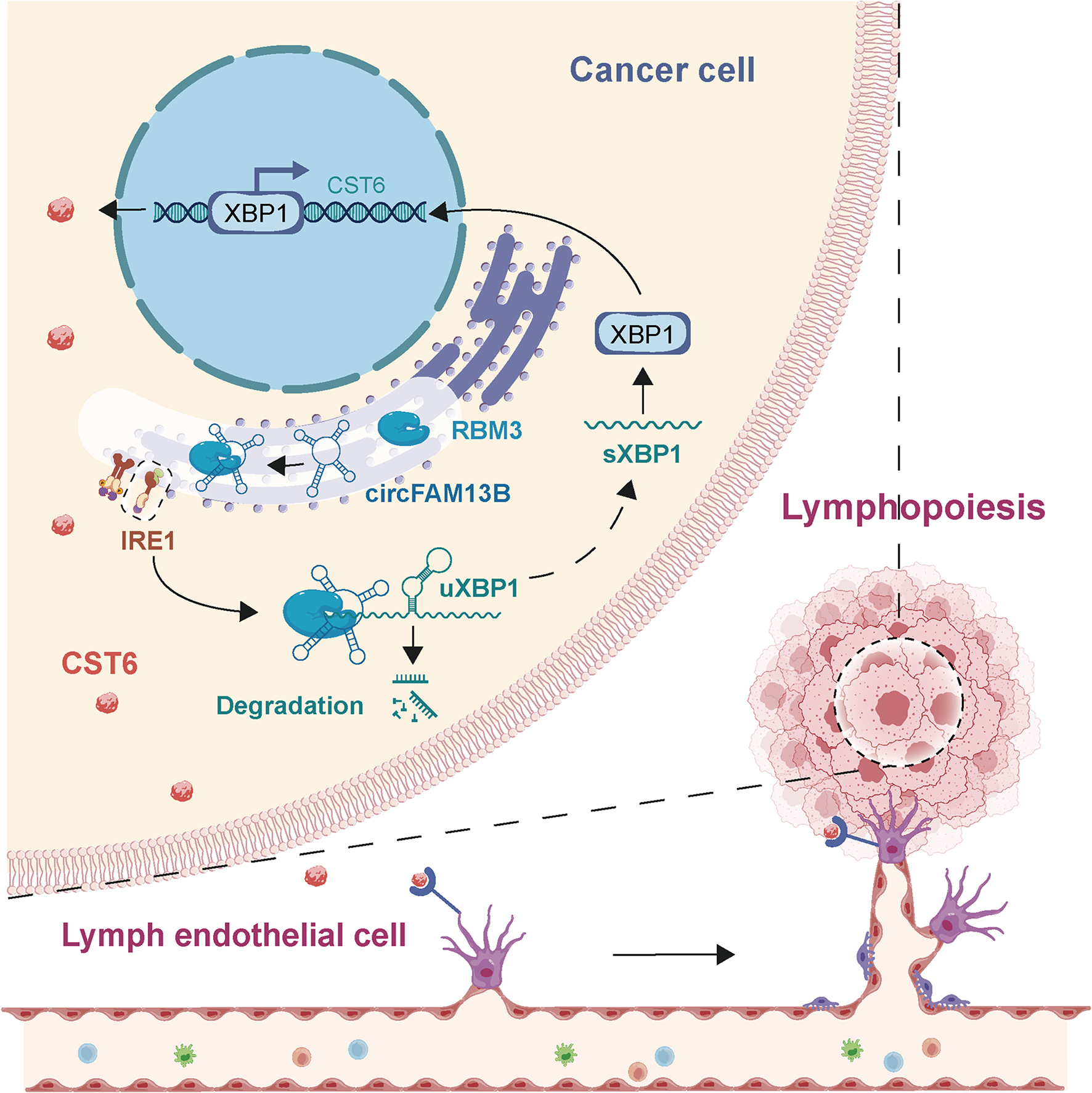

## Introduction

Nasopharyngeal carcinoma (NPC) is a malignant tumor that originates from the nasopharyngeal epithelium and is geographically predominant in Southeast Asia, North Africa and the Middle East [1]. Histologically, NPC are mainly undifferentiated or poorly differentiated squamous cell carcinomas that are sensitive to radiotherapy and chemotherapy. Radiotherapy is currently the curative treatment for NPC [2], with a local control rate of > 90%. However, NPC is also prone to lymph node metastasis [3, 4]. More than 80% of patients have cervical lymph node enlargement at the time of initial diagnosis, leading to treatment failure in 15-42% of patients due to early lymph node metastasis [5]. Therefore, it is crucial to elucidate the molecular mechanisms underlying lymph node metastasis in NPC to develop more effective treatment strategies.

Circular RNAs (circRNAs) are a class of single-stranded circular RNA molecules primarily formed through splicing [6]. Although their efficiency is lower than that of linear splicing, circRNAs are highly stable once they form and continuously accumulate in cells [7]. Accumulating evidence suggests that circRNAs play various physiological and pathological roles in tumor development, including enhancing tumor cell stemness, regulating the cell cycle, inhibiting apoptosis, and promoting autophagy, angiogenesis, and proliferation [8–12]. Several studies have indicated an association between circRNAs and tumor lymphatic metastasis and angiogenesis [13–15]. circRNAs can function as competitive endogenous RNAs (ceRNAs) or protein-coding RNAs or interact with RNA-binding proteins to regulate the expression of genes involved in tumorigenesis and progression [8, 9, 16]. However, the specific functions and mechanisms of circRNAs in NPC lymphatic metastasis are still not fully understood.

Unfolded protein response (UPR) is a cellular stress response mechanism designed to maintain protein homeostasis in endoplasmic reticulum (ER). When a large amount of unfolded or misfolded proteins accumulates in ER, ER stress is triggered, thereby activating the UPR to restore the normal folding and function of proteins [17]. However, when external factors persist (including hypoxia, accumulation of reactive oxygen species, low pH, inactivation of tumor suppressor genes, or activation of oncogenes), the three ER transmembrane proteins (ATF6/IRE1α/PERK) are activated, leading to a cascade of stress responses and sustained activation of ER stress [17–19]. This subsequently contributes to tumor initiation and progression. Exploring the ER stress signaling pathway can provide a clearer understanding of the mechanisms underlying lymphatic metastasis in NPC.

Here, we identified circFAM13B, which is located in ER, is downregulated in NPC patients with lymph node metastasis. circFAM13B enhances the interaction between RNA-binding motif protein 3 (RBM3) and the critical ER signaling molecule precursor uXBP1 mRNA, promoting its degradation, leading to a decrease in the nuclear translocation of the transcription factor sXBP1, and subsequently inhibiting cytokine CST6 transcription. This inhibition leads to the suppression of invasion, metastasis of NPC cells and lymphatic vessel formation. Taken together, our data reveal a novel mechanism underlying the lymph node metastasis, and suggest promising therapeutic targets in NPC.

## Results

### CircFAM13B is expressed at low levels in lymphatic metastatic NPC tissues

**Fig. 1:**
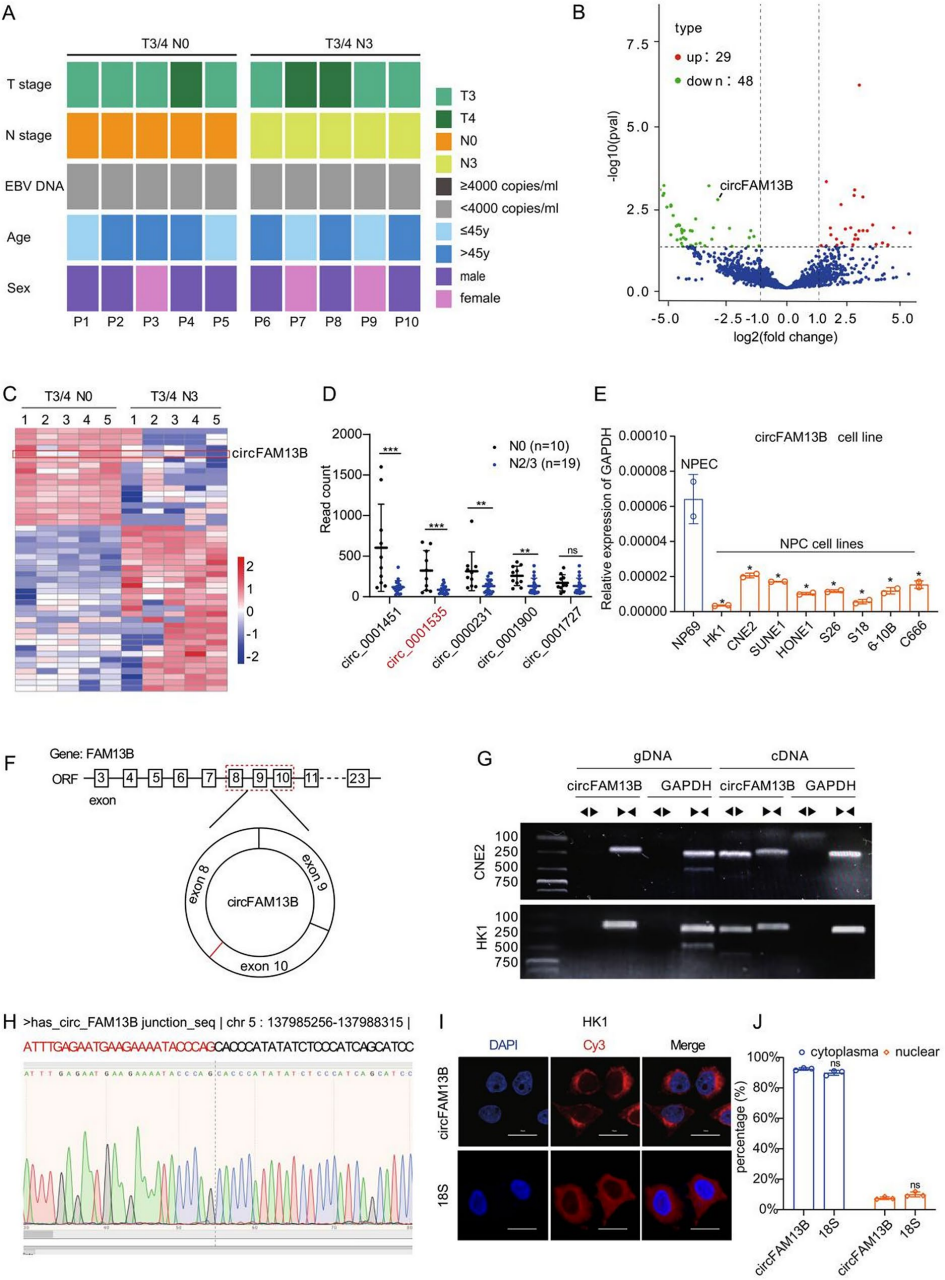
CircFAM13B is downregulated in lymphatic metastatic NPC tissues. **A**. Comparison of baseline characteristics between the two sequencing cohorts. **B**-**C**. Volcano plot (**B**) and heatmap (**C**) showing the expression of circular RNAs in transcriptome sequencing data. The x-axis in volcano plots depicts log2-transformed fold changes (FC) of Rs versus NRs. The vertical dashed lines represent the log2-transformed FC thresholds of 1 or − 1. The horizontal dashed line represents the two-sided DESeq2-adjusted *p* value threshold of 0.05. **D**. The sequencing results of the expanded samples demonstrated differential expression of circRNAs between the N0 group (*n* = 10) and the N2/3 group (*n* = 19).** E**. qPCR results of circFAM13B in nasopharyngeal epithelial cells and NPC cells. **F**. Illustrative diagram of circFAM13B circularization. **G**. Agarose gel electrophoresis of PCR products using convergent and divergent primers in genomic DNA and total RNA cDNA. **H**. Sanger sequencing peaks of circFAM13B PCR products using divergent primers. **I**-**J**. Fluorescence in situ hybridization of circFAM13B in nasopharyngeal carcinoma cells, with 18S as a cytoplasmic RNA control. The error bars represent standard deviations of three independent experiments. Scale bar: 20 μm. *p < 0.05, **p < 0.01 and ***p < 0.001

To identify the key molecules involved in the lymphatic metastasis of NPC, primary tumor tissues without lymph node metastasis (five cases, clinical stage T3/4N0M0) and with lymph node metastasis (five cases, clinical stage T3/4N3M0) were collected for transcriptome sequencing. The two groups were well-matched in terms of baseline characteristics (Fig. 1A). Bioinformatic analysis of the sequencing results revealed significant differences in the expression of multiple circular RNAs between the two groups (Fig. 1B, C). To validate the sequencing results, 10 cases of N0 and 19 cases of N2/3 NPC tissues were collected to detect differentially expressed circRNA expression. The results were consistent with the sequencing results, circFAM13B was highly expressed in the N0 group (Fig. 1D). Additionally, circFAM13B was downregulated in NPC cell lines compared with NPEC NP69, suggesting its potential inhibitory role in cancer cell lymphatic metastasis (Fig. 1E). Bioinformatics analysis revealed that circFAM13B originates from the parent gene FAM13B, which has 23 exons, with exons 3–23 constituting the open reading frame (ORF). Exons 8, 9, and 10 were circularized to form circFAM13B (Fig. 1F). Genomic DNA (gDNA) and total RNA were extracted from the NPC cells CNE2 and HK1, and reverse transcription PCR was performed to target the circFAM13B circularization site using specific forward and reverse primers for circFAM13B and GAPDH. The results showed the presence of circFAM13B open products only in reverse-transcribed cDNA from total RNA, suggesting circularization (Fig. 1G). Sanger sequencing of the open products confirmed sequence consistency with the circularization site (Fig. 1H). Fluorescence in situ hybridization (FISH) results showed that circFAM13B mainly resides in the cytoplasm, particularly at the periphery of the cell nucleus (Fig. 1I, J).

### CircFAM13B inhibits the metastasis of NPC cells in vitro and in vivo

**Fig. 2:**
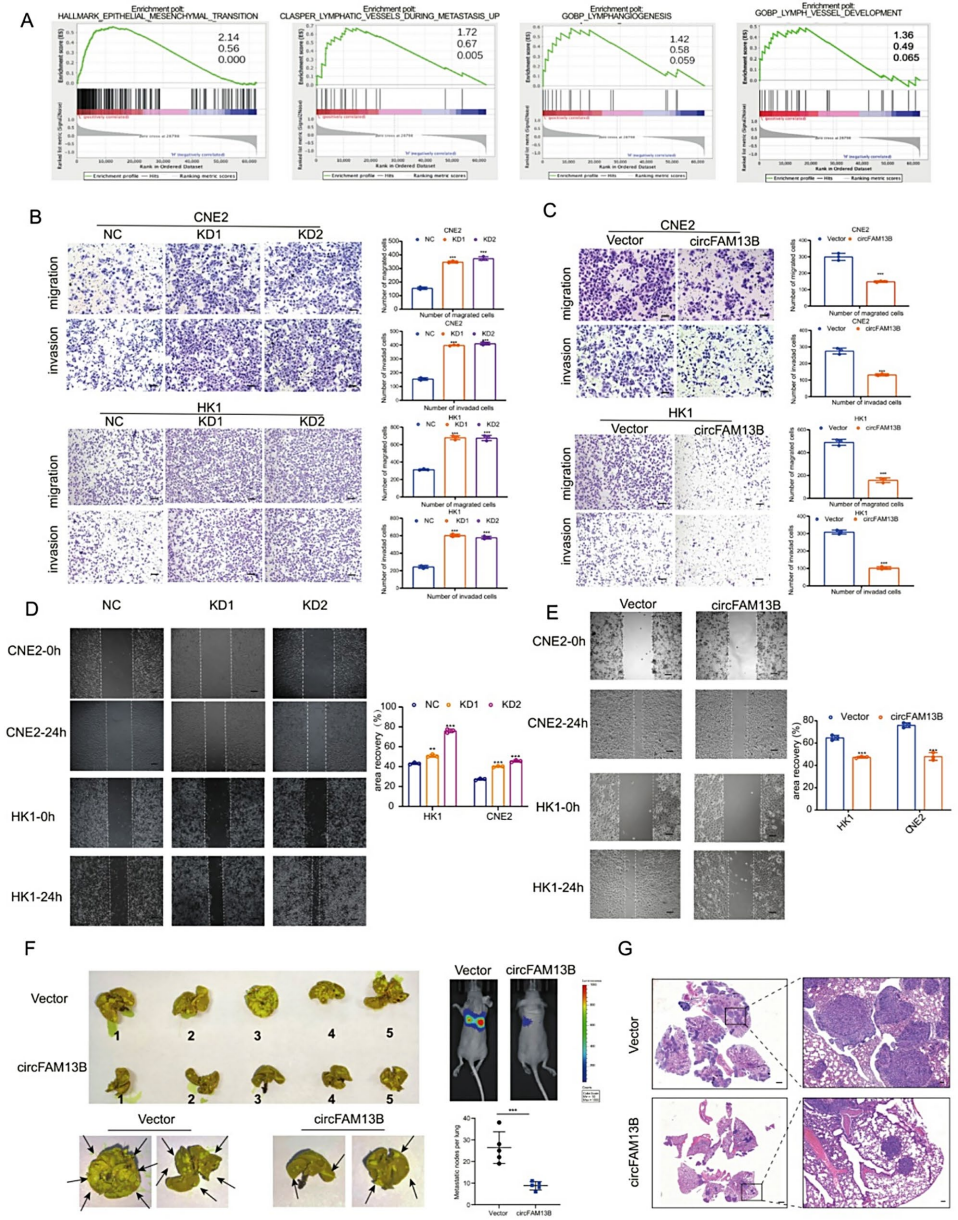
Knockdown or overexpression of circFAM13B affects invasion and migration of NPC cells in vitro and in vivo. **A**. Gene Set Enrichment Analysis (GSEA) of clinical samples sequencing results. **B**, **C**. Invasion (upper row) and migration (lower row) assays of circFAM13B knockdown (**B**) and overexpression (**C**) stable cells. Scale bar: 100 μm. **D, E**. Scratch wound healing assay of circFAM13B knockdown (**D**) and overexpression (**E**) stable cells. Scale bar: 100 μm. **F**. Representative gross images, fluorescent images, and statistical analysis of lung metastatic nodules in mice after intravenous injection, picric acid staining, and formalin fixation. Yellow nodules represent metastatic tumors. **G**. **H**&**E** staining of lung sections after paraffin embedding. Scale bar: 1.25 mm (left) or 100 μm (right). The error bars represent standard deviations of three independent experiments. *p < 0.05, **p < 0.01 and ***p < 0.001

Gene Set Enrichment Analysis (GSEA) of the clinical samples sequencing data showed that the downregulation of circFAM13B was associated with pathways related to epithelial-mesenchymal transition (EMT), lymphangiogensis and lymph vessel development, indicating that circFAM13B may affect tumor cell invasion and metastasis (Fig. 2A). To validate the function of circFAM13B in NPC, we successfully constructed stable cell lines of circFAM13B overexpression and knockdown (Supplementary Fig. 1A). In addition, western blotting result demonstrates that the expression of FAM13B protein was not affected after overexpression or knockdown of circFAM13B (Supplementary Fig. 1B). Next, in vitro transwell assays (Fig. 2B, C) and wound healing assays (Fig. 2D, E) were performed. Consistent with the GSEA results, circFAM13B knockdown significantly promoted the migratory and invasive abilities of NPC cell lines, whereas overexpression of circFAM13B inhibited the invasion and migration. These findings demonstrated the inhibitory effect of circFAM13B on the invasion and metastasis of NPC cells in vitro. Additionally, the effect of circFAM13B on the proliferation and colony formation ability of NPC cells was assessed. CCK8 experiments showed that the proliferation of circFAM13B overexpressed stable cells were similar with that of the control cells, circFAM13B knockdown did not significantly affect proliferation of NPC cells (Supplementary Fig. 1C, D). Furthermore, colony formation assays showed no difference in both the size and the number of colonies between the knockdown, overexpression and respective control groups, indicating that circFAM13B does not affect the proliferative capacity of NPC cells (Supplementary Fig. 1E, F). To validate the in vivo function of circFAM13B, a mouse lung metastasis model was established using tail vein injection of stable overexpression cell lines. After four weeks, the mice were examined for lung metastasis. The results showed a significant reduction in the number of metastatic lung nodules in the group overexpressing circFAM13B (Fig. 2F). Histological analysis with hematoxylin and eosin (HE) staining confirmed the presence of metastatic tumor nodules in lung tissues (Fig. 2G).

### CircFAM13B inhibits lymphangiogenesis and lymphatic metastasis of NPC

**Fig. 3:**
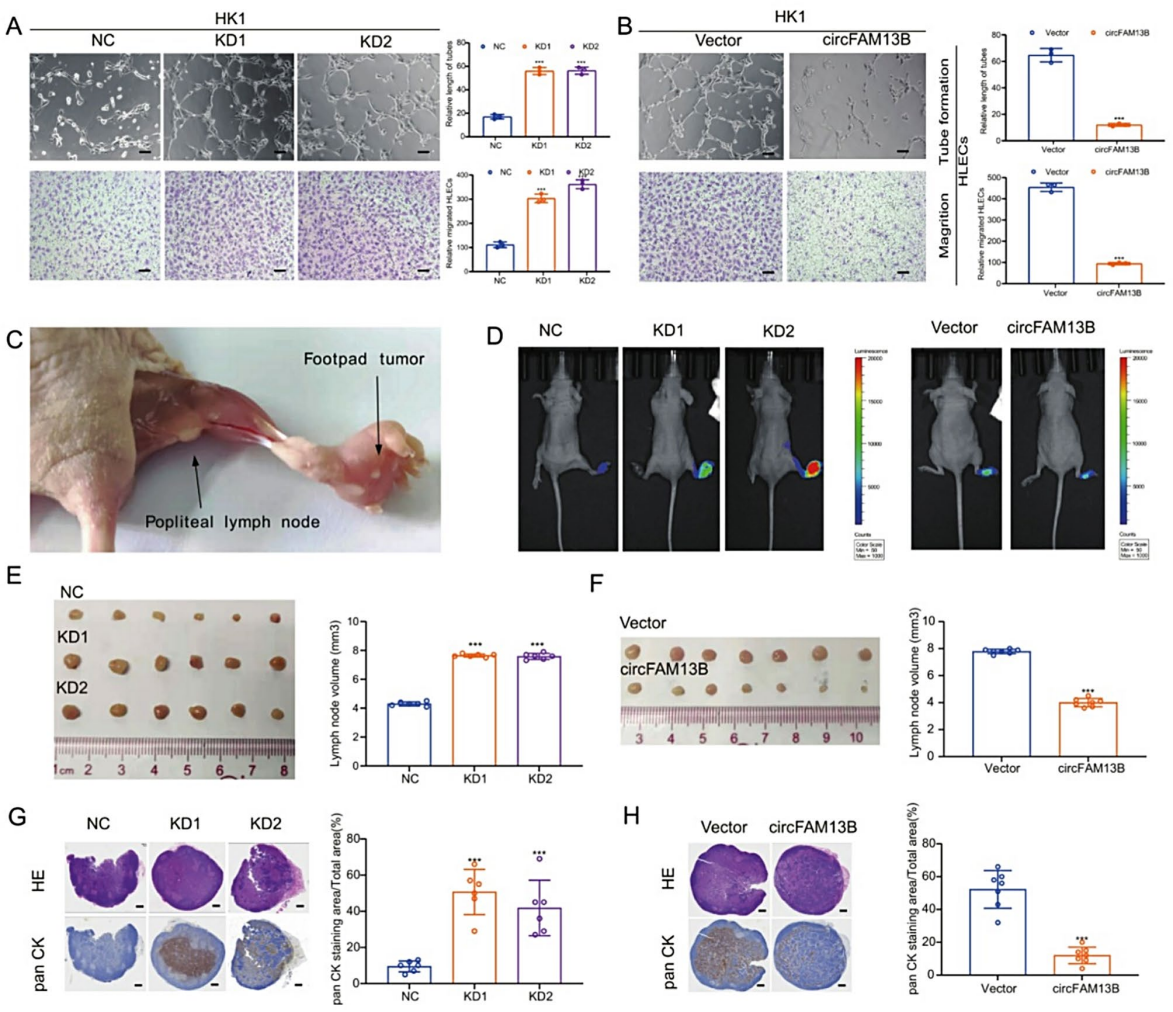
CircFAM13B inhibits lymphangiogenesis and lymphatic metastasis. **A**, **B**. Tube formation assays of lymphatic endothelial cells in CM from circFAM13B knockdown (**A**) and overexpression (**B**) NPC cells. Scale bar: 100 μm. **C**. Schematic representation of the mouse footpad injection and popliteal lymph node metastasis model. **D**. In vivo fluorescence imaging of footpad and popliteal lymph nodes in mice with circFAM13B knockdown or overexpression stable cells. **E**, **F**. Statistics of popliteal lymph node volumes in mice with circFAM13B knockdown (**E**) and overexpression (**F**) stable cells. **G**, **H**. **H**&**E** staining (upper row) and pan-CK immunohistochemical staining (lower row) of popliteal lymph nodes in mice with circFAM13B knockdown (**G**) and (**H**) stable cells. Scale bar: 300 μm. The error bars represent standard deviations of three independent experiments. *p < 0.05, **p < 0.01 and ***p < 0.001

Since the GSEA results suggested that the downregulation of circFAM13B was associated with lymphangiogenesis, we collected conditioned medium (CM) from stable NPC cell lines and co-cultured with lymphatic endothelial cells. The results of the lymphatic tube formation experiment showed that the CM of circFAM13B knockdown cells significantly promoted lymphatic tube formation compared to control cells (Fig. 3A). Conversely, CM from circFAM13B overexpression cell lines inhibited lymphatic tube formation (Fig. 3B).

For the in vivo experiments, we used a mouse footpad injection popliteal lymph node metastasis model (Fig. 3C). Popliteal lymph node metastases were observed after 4–6 weeks since injection. Consistent with the in vitro results, circFAM13B knockdown significantly promoted tumor cell metastasis to the popliteal lymph nodes, which was characterized by an increased volume of the popliteal lymph nodes and an increased proportion of tumor cells within the lymph nodes (Fig. 3D, E, G). In contrast, the overexpression of circFAM13B significantly inhibited tumor cell metastasis to the popliteal lymph nodes (Fig. 3D, F, H). Taken together, these results suggest that circFAM13B is a key molecule associated with lymphatic metastasis in NPC.

### CircFAM13B specifically localizes in the ER and regulates key signaling pathways of ER stress

**Fig. 4:**
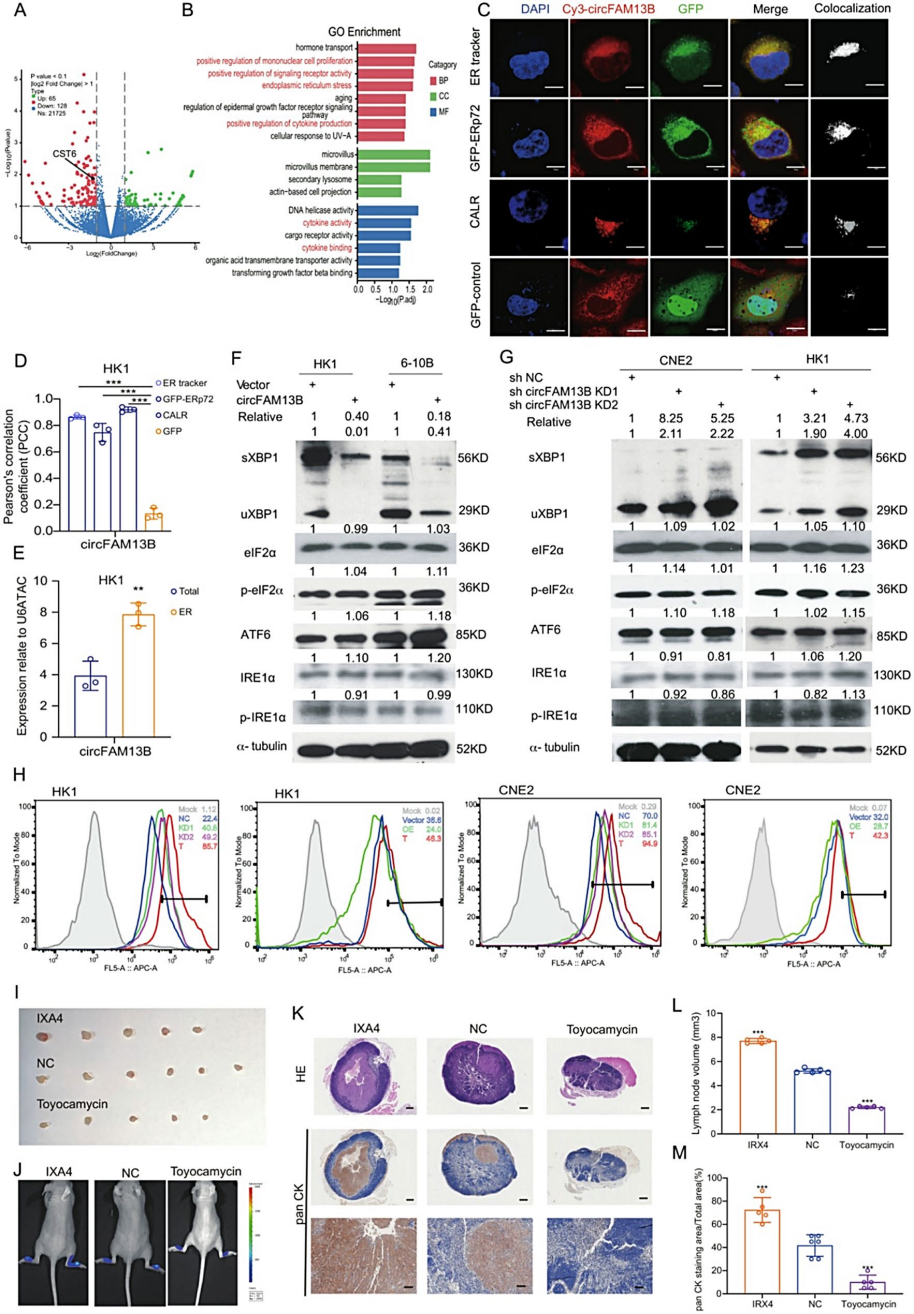
CircFAM13B regulates the key signaling molecule XBP1 in endoplasmic reticulum stress. **A**. Volcano plot of differentially expressed genes (DEGs) in circFAM13B stable cell line transcriptome sequencing. **B**. GO enrichment analysis of DEG. **C**. Immunofluorescence staining of ER-related proteins and in situ hybridization of circFAM13B showing the localization of circFAM13B in the endoplasmic reticulum. Scale bar: 10 μm. **D**. Quantification of colocalization index by Pearson’s correlation coefficient (PCC) of Fig. 4C. Data represent mean ± SEM (*n* = 3). **E**. qRT-PCR analysis of circFAM13B expression in total and endoplasmic reticulum extracts. **F**, **G**. The signaling molecules related to endoplasmic reticulum stress were examined in circFAM13B overexpression (**F**) and knockdown (**G**) stable cells by immunoblotting. Grayscale analysis and relative values of the target bands (a-tubulin was used as a loading control) were labeled above the bands. **H**. Flow cytometry analysis of XBP1 expression in circFAM13B knockdown and overexpression stable cells. The positive control (T) was 300 nM Thapsigargin treated for 18 h (red) and the negative control group (cells incubated with only the secondary antibody) was also included (gray). **I**. Representative images of popliteal lymph nodes in mouse injected with NC, IXA4, and Toyocamycin respectively. **J**. In vivo fluorescence imaging of footpads and popliteal lymph nodes in mouse groups injected with NC, IXA4, and Toyocamycin respectively. **K**. Representative images of HE staining (upper row) and PAN-CK immunohistochemical staining (middle lower row) of popliteal lymph nodes of mice injected with NC, IXA4, and Toyocamycin respectively. Scale bars in the upper and middle rows: 100 μm; scale bars in the lower row: 300 μm. **L**. Histogram analysis of the volume (**L**) and PAN-CK immunohistochemical staining scores (**M**) of popliteal lymph nodes in mouse groups injected with NC, IXA4, and Toyocamycin respectively. The error bars represent standard deviations of three independent experiments. *p < 0.05, **p < 0.01 and ***p < 0.001

To explore the molecular mechanism by which circFAM13B inhibits tumor lymphatic metastasis, we conducted transcriptome sequencing of HK1 stable cell lines. Overexpression of circFAM13B altered the expression of multiple genes (Fig. 4A). Gene Ontology Analysis (GO) of differentially expressed genes revealed that circFAM13B was associated with cytokine activity, receptor activity and endoplasmic reticulum (ER) stress (Fig. 4B). Therefore, we speculated that circFAM13B may be involved in ER function. To validate this hypothesis, we label the ER with an ER tracker and EGFP-tagged ER-specific protein, Erp72, and in situ hybridization of circFAM13B was performed in same cells. The results showed the co-localization of circFAM13B with the ER tracker and EGFP-Erp72, while no colocalization was observed in control GFP group. We then further performed additional experiments using calreticulin (CALR) as a new ER marker and captured images using a state-of-the-art high-resolution confocal microscope (AX NSPAPC), with a colocalization index of 0.94, which was consistent with previous results (Fig. 4C-D). In addition, following the isolation of the endoplasmic reticulum (ER) using an ER extraction kit and subsequent RNA extraction, the results demonstrated that circFAM13B was significantly enriched in the ER fractions compared to total RNA (Fig. 4E). These results suggest that circFAM13B is indeed localized in ER. To test if circFAM13B affects ER stress, the three key signaling pathways involved in ER stress, that is IRE1-XBP1, PERK-p-eIF2a, and ATF6, were detected in stable cell lines. Western blotting (WB) analysis showed that overexpression of circFAM13B did not affect the expression of ATF6 or phosphorylation of eIF2a, but decreased the expression of uXBP1 (full-length) and sXBP1 (spliced) (Fig. 4F). In contrast, circFAM13B knockdown upregulated uXBP1 and sXBP1 expression (Fig. 4G). Flow cytometric analysis of XBP1 in stable cell lines with circFAM13B knockdown or overexpression showed similar results (Fig. 4H). Additionally, RNA SCOPE of circFAM13B and XBP1 immunohistochemistry were performed using tissue microarrays from 74 cases. High XBP1 expression was observed in NPC tissues, and there was a significant negative correlation between circFAM13B expression and XBP1 (Supplementary Fig. 2A, B). For the in vivo popliteal lymph node metastasis experiments, XBP1 inhibitor Toyocamycin (0.5 mg/kg intraperitoneal injection twice a week for two weeks) and XBP1 activator IXA4 (0.5 mg/kg intraperitoneal injection twice a week for two weeks) was intraperitoneally injected respectively. Compared with the control group, the IXA4 group significantly promoted the metastasis of tumor cells to popliteal lymph node. On the contrary, the Toyocamycin group significantly inhibited the metastasis of tumor cells to popliteal lymph nodes (Fig. 4I-M). These results suggest that circFAM13B specifically localizes to the ER and downregulates the key signaling molecule XBP1 during ER stress.

### CircFAM13B enhances the interaction between RBM3 and uXBP1, promotes degradation of uXBP1

**Fig. 5:**
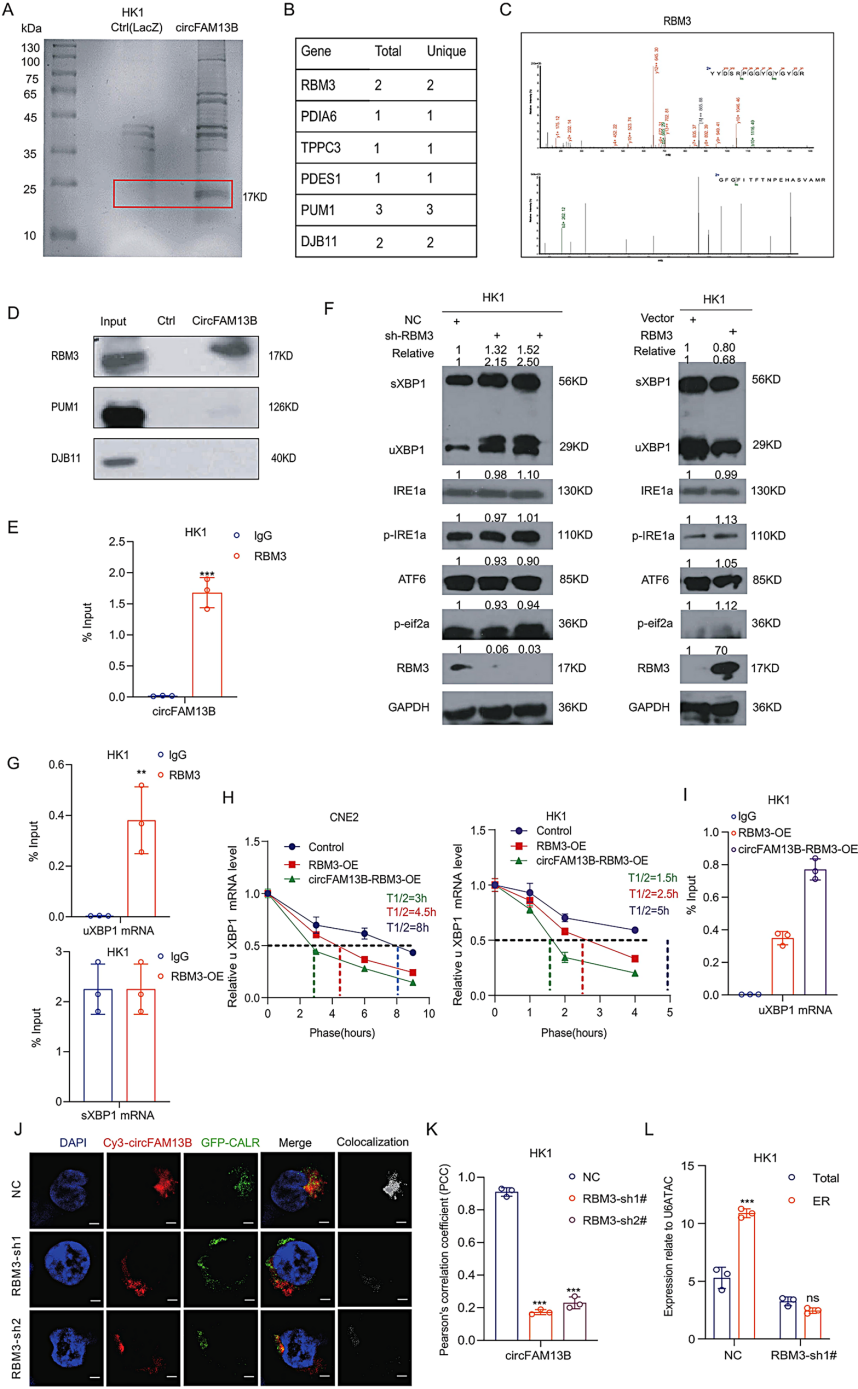
CircFAM13B enhances the interaction between RBM3 and uXBP1 mRNA, and promotes uXBP1’s degradation. **A**. Silver-stained gel after RNA pull-down and protein separation. LacZ probe as negative control. **B**. Number of peptides related to endoplasmic reticulum-associated proteins in proteomic results. **C**. RBM3 peak diagram in mass spectrometry results. **D**. Verification of mass spectrometry results by RNA pull-down. **E**. Reverse immunoprecipitation (RIP) assays confirming the interaction between RBM3 protein and circFAM13B. **F**. The signaling molecules related to endoplasmic reticulum stress were examined in RBM3 overexpression and knockdown stable cells by immunoblotting (*n* = 3). **G**. RIP assays showing the interaction of RBM3 protein with uXBP1 but not sXBP1. H. circFAM13B enhances the interaction between RBM3 and uXBP1 mRNA, promoting degradation after actinomycin **D**. conduction. The half-life of uXBP-1 mRNA was calculated and displayed as T1/2 values (*n* = 3). **I**. The RBM3-RIP assay of uXBP1 mRNA based on overexpression of circFAM13B. The error bars represent standard deviations of three independent experiments. **J**. Immunofluorescence staining and in situ hybridization showing the localization of circFAM13B in the endoplasmic reticulum following RBM3 knockdown. Scale bar: 10 μm. **K**. Quantification of colocalization index by Pearson’s correlation coefficient (PCC. Data represent mean ± SEM (*n* = 3). **L**. qRT-PCR analysis of circFAM13B expression in total and endoplasmic reticulum extracts following RBM3 knockdown. *p < 0.05, **p < 0.01 and ***p < 0.001

To further investigate the mechanism by which circFAM13B affects ER stress, we conducted RNA pull-down experiments with a circFAM13B-specific probe. The results indicated that the proteins interacting with circFAM13B included several molecules which were associated with ER stress, such as DNAJB11, RBM3, and PDIA6 (Fig. 5A-C). RBM3 drew our attention because of its high score, it is a member of the glycine-rich RNA-binding protein family and encodes a protein with one RNA recognition motif (RRM) domain. Expression of this gene is induced by cold shock and low oxygen tension [20–22]. Western blotting validated the mass spectrometry results and confirmed the interaction between circFAM13B and RBM3 (Fig. 5D). Additionally, we performed RNA immunoprecipitation (RIP) experiments using RBM3 antibodies and confirmed the interaction between RBM3 and circFAM13B (Fig. 5E). These data demonstrate that circFAM13B interacts with RBM3.

Does RBM3 affect the expression of XBP1? We found that RBM3 overexpression led to a decrease in the expression of uXBP1 and sXBP1, whereas RBM3 knockdown upregulated the expression of uXBP1 and sXBP1 (Fig. 5F). RIP experiments revealed that RBM3 binds to uXBP1 but not sXBP1 mRNA (Fig. 5G). Previous studies have shown that RBM3 can bind to the 3′UTR to impact mRNA stability [21]. Therefore, we conducted actinomycin D mRNA stability assays and found that the half-life of uXBP1 mRNA decreased when RBM3 was overexpressed. This result indicated that RBM3 promoted uXBP1 mRNA degradation. Upon overexpression, CircFAM13B exerts a dual influence: it significantly shortens the half-life of uXBP1 mRNA (Fig. 5H) and enhances the interaction between RBM3 and uXBP1 as evidenced by RIP (Fig. 5I). These data together suggest that circFAM13B facilitates RBM3-mediated degradation of uXBP1 and downstream sXBP1 mRNA.

### RBM3 is essential for circFAM13B ER localization

Our prior findings had pinpointed RBM3 as a binding protein for circFAM13B (Fig. 5A-I). Subsequently, we delved into its subcellular localization. Immunofluorescence staining with an RBM3 - specific antibody and ER tracker labeling unveiled that RBM3 predominantly resided in the cytoplasm and largely co-localized with the ER (Supplementary Fig. 3A), mirroring the distribution pattern of circFAM13B. CircFAM13B knockdown failed to alter RBM3 localization (Supplementary Fig. 3B), indicating that RBM3’s localization is independent of circFAM13B. To explore whether RBM3 mediates the ER localization of circFAM13B, in situ hybridization combined with CALR labeling in RBM3 - knockdown stable cells demonstrated a significant reduction in the colocalization of circFAM13B with the ER following RBM3 knockdown (Fig. 5J-K). Consistently, ER fractionation followed by RNA extraction showed that RBM3 depletion abolished the enrichment of circFAM13B in the ER fraction (Fig. 5L). Taken together, these results support the notion that RBM3 is essential for the ER localization of circFAM13B, while RBM3 itself localizes to the ER is independently of circFAM13B.

### sXBP1 regulates the transcription of the downstream key factor CST6, thereby affecting lymphangiogenesis

**Fig. 6:**
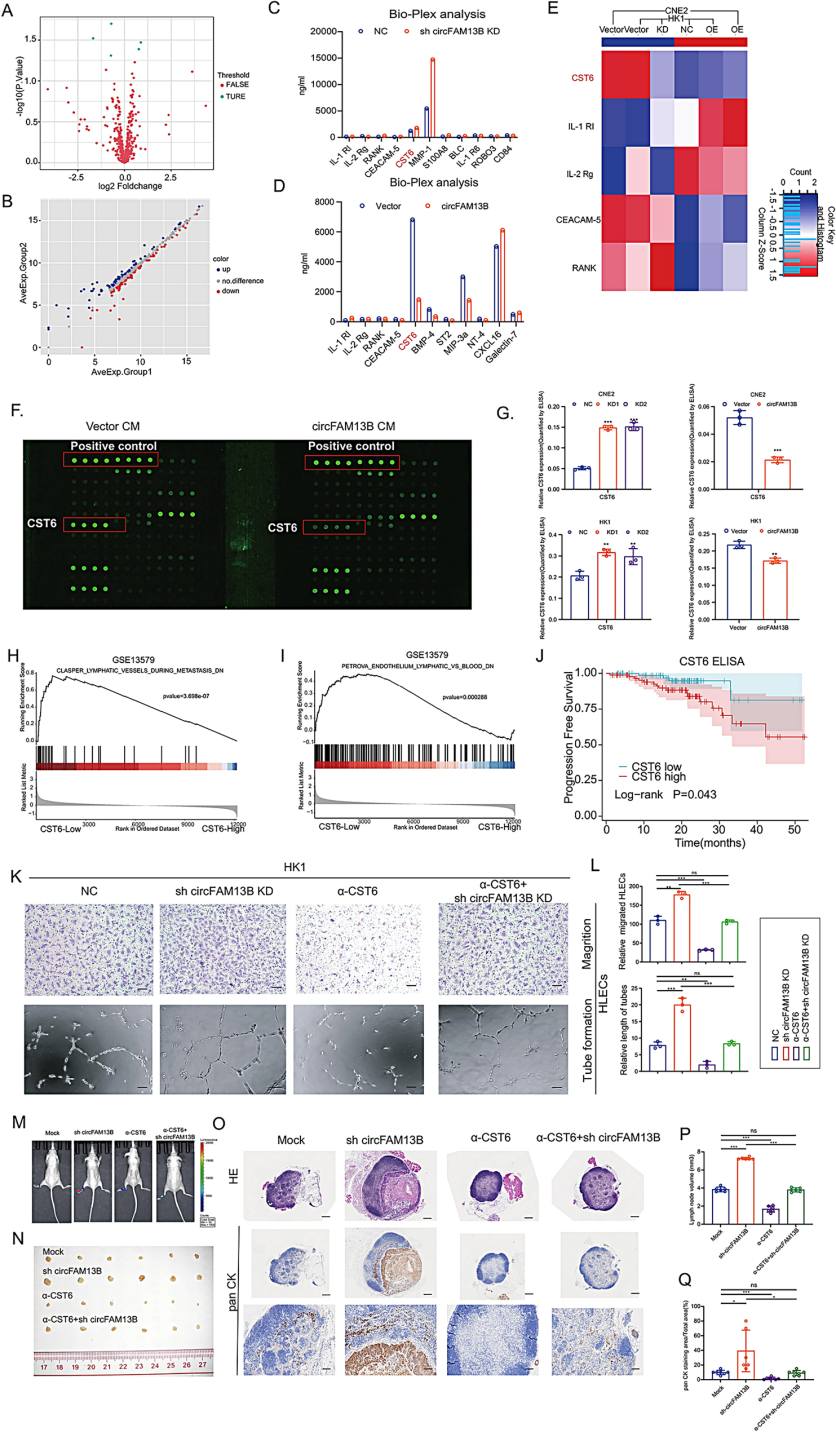
CST6 promotes lymph node metastasis of NPC cells. **A**, **B**. Volcano plots (**A**) and Scatter plot (**B**) of CM cytokine chip array in circFAM13B stable cell. The TRUE are fold change greater than 1.2 or less than 0.83 and *p* value less than 0.05. **C**, **D**. Bar plot of differentially expressed proteins in CM of stable circFAM13B knockdown (**C**) and overexpression (**D)** cells. **E**. Heatmap of differentially expressed proteins in circFAM13B stable cells. **F**. Original image of cytokine chip. **G**. CST6 ELISA in condition medium of circFAM13B overexpression and knockdown cells. **H**-**I**. GSEA analysis of NPC public data. **J**. Kaplan–Meier analysis of progress-free survival (PFS) based on CST6 serum expression in 168 NPC patients. **K**-**L**. Representative images (**K**) and quantification (**L**) of transwell migration and lymphatic vessel formation of HLECs treated with indicated CM. Scale bar: 100 μm. **M, N**. In vivo fluorescence imaging (M) and images (**N**) of popliteal lymph node metastasis with CST6-neutralizing antibody. **O**. Representative images of HE staining (top row) and PAN-CK immunohistochemical staining (middle and bottom row) of popliteal lymph node metastases with a CST6-neutralizing antibody. Scale bar for upper and middle rows: 100 μm; scale bar for lower row: 300 μm. **P**, **Q**. Histogram analysis of popliteal lymph node volume (**P**) and PAN-CK immunohistochemical staining score (**Q**) with a CST6-neutralizing antibody. The error bars represent standard deviations of three independent experiments. *p < 0.05, **p < 0.01 and ***p < 0.001

Previous results showed that the CM of the NPC stable cell lines affected lymphangiogenesis in vitro; indicate that circFAM13B may inhibit lymphatic metastasis via affecting cytokine expression. Cytokine microarray consisted of 440 cytokines was used and three cytokines, RANK, CEACAM-5, and CST6, were highly expressed in the circFAM13B knockdown CM and lowly expressed in the circFAM13B overexpressed CM (Fig. 6A-E).

Among the differentially expressed cytokines, CST6 showed the most significant difference (adj. *P* = 0.001) (Fig. 6F), which was consistent with the stable cell lines transcriptomic analysis (Supplementary Fig. 4B). As well as GO and KEGG analysis of transcriptome sequencing and cytokine array suggests that circFAM13B is associated with cytokine-cytokine receptor interaction (Supplementary Fig. 4A-E). Enzyme-Linked Immunosorbent Assay (ELISA) of CST6 with circFAM13B stable cell lines’ CM validated the cytokine microarray results, circFAM13B was negatively correlated with secreted CST6 (Fig. 6G).

The GSEA analysis of GEO public database (GSE13579) indicated that CST6 is associated with lymphatic vessel formation during tumor metastasis (Fig. 6H-I). Serum samples from 168 NPC patients were collected (Supplementary Table 1) and performed CST6 ELISA. The results showed that high CST6 expression was negatively correlated with progression-free survival in patients (Fig. 6J).

To explore the inhibitory function of circFAM13B is dependent on CST6, we collected four sets of CM, including control CM, circFAM13B knockdown CM, control CM plus CST6 neutralizing antibody and circFAM13B knockdown CM plus CST6 neutralizing antibody, in co-cultured with lymphatic endothelial cells. The results showed that circFAM13B knockdown promoted tube formation and migration of lymphatic endothelial cell. CST6 neutralizing antibody partial reduced tube formation and migration of tube formation and migration group (Fig. 6K, L). Furthermore, CST6 neutralizing antibody significantly reduced circFAM13B-transduced tumor burden in lymph nodes (Fig. 6M–Q), indicating that blocking CST6 signaling abrogates circFAM13B-induced lymphatic metastasis in vivo.

These results indicate that CST6 can significantly promote the tube formation and migration ability of lymphatic endothelial cells, and the function of circFAM13B in inhibiting lymphatic metastasis depends on CST6.

### XBP1 acts as a transcription factor to regulate the expression of CST6

**Fig. 7:**
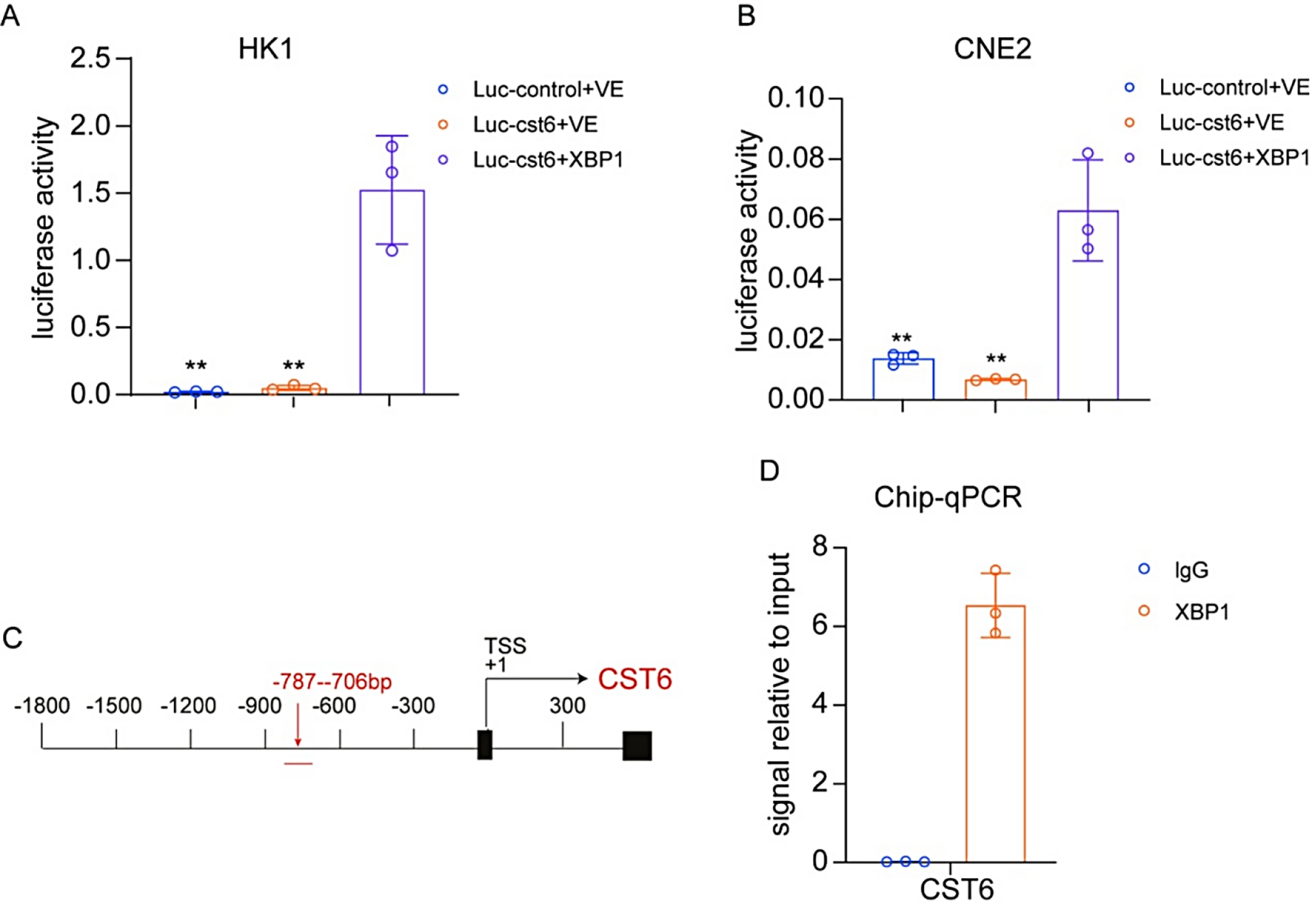
XBP1 regulates CST6 expression as a transcription factor. **A**-**B**. Dual-luciferase assay confirming XBP1 activation of the CST6 promoter. **C**. Schematic diagram of potential XBP1 binding sites and primer location in the CST6 promoter. **D**. ChIP assay after truncation of the CST6 promoter reveals significant binding between − 900–600 bp. The error bars represent standard deviations of three independent experiments. *p < 0.05, **p < 0.01 and ***p < 0.001

During ER stress, uXBP1 splicing becomes sXBP1, which translates proteins into the nucleus and acts as transcription factors to regulate the expression of downstream genes [23, 24]. Since we observed that circFAM13B can promote the expression of sXBP1 and at the same time up-regulate the expression of cytokine CST6, it is speculated that sXBP1 may regulate the transcription of CST6 as a transcription factor. To test this theory, we conducted dual-luciferase experiments and found that sXBP1 could activate the promoter of CST6 (Fig. 7A, B). In addition, Chromatin Immunoprecipitation (ChIP) experiments showed that the upstream − 900–600 bp region (primers designed in the − 787–706 bp region) of the CST6’s transcription start site was the binding site for sXBP1 (Fig. 7C-D). These data demonstrate that circFAM13B regulates the transcriptional regulation of CST6 by inhibiting the key signaling molecule XBP1 during ER stress, and affecting lymphangiogenesis.

### Clinical specimen analysis of the relationship between high circFAM13B expression and prognosis of NPC patients

**Fig. 8:**
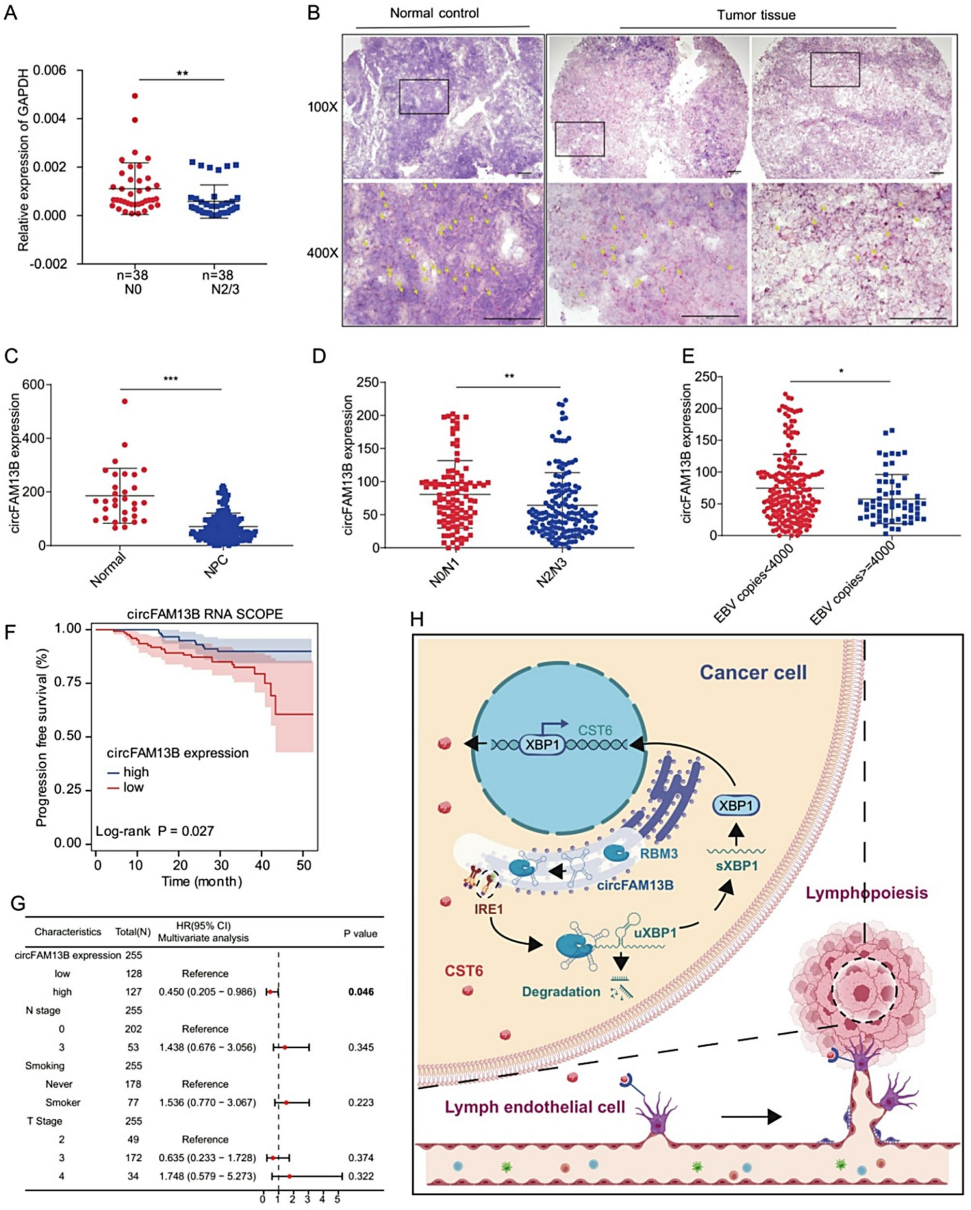
Low expression of circFAM13B is associated with poor prognosis in NPC. **A**. qRT-PCR of circFAM13B expression in non-lymph node metastasis and lymph node metastasis NPC patients. **B**. RNA SCOPE of circFAM13B in normal and NPC patients. scale bar: 100 μm. **C**-**E**. Subgroup analysis of normal individuals and tumor patients, NPC patients with different N stages and varying EBV expression based on circFAM13B expression. **F**. Survival analysis demonstrating high circFAM13B expression is associated with better prognosis in NPC patients. **G**. Forest plot of multivariate analysis based on progression-free survival according to circFAM13B expression and baselines characters. **H**. Schematic diagram showing the mechanism by which circFAM13B inhibits NPC lymph node metastasis through enhancing the interaction between RBM3 and uXBP1 mRNA, promoting uXBP1’s degradation and inhibiting the secretion of the downstream cytokine CST6. *p < 0.05, **p < 0.01 and ***p < 0.001, CI: confidence interval

To validate the sequencing results, we collected NPC tissues from 38 patients without lymph node metastasis (N0) and 38 with lymph node metastasis (N2/3). Total RNA was extracted and circFAM13B expression was detected. The results showed that circFAM13B was highly expressed in the N0 group, which was consistent with the sequencing results (Fig. 8A). Additionally, we performed RNA SCOPE experiments using tissue microarrays from 255 patients (Supplementary Table 2). The results revealed that circFAM13B, a tumor suppressor molecule, was highly expressed in normal tissues (Fig. 8B, C). Subgroup analyses showed that circFAM13B was highly expressed in the N0/N1 group and the group with EBV copies < 4000 (Fig. 8D, E,). Survival analysis demonstrated that patients with high circFAM13B expression had a better prognosis in NPC (Fig. 8F). On multivariate analysis, circFAM13B expression was an independent prognostic factor for progression-free survival (Supplementary Table 3, Fig. 8G). Compared to patients with low expression, patients with high expression of circFAM13B have a 55% reduced risk of disease progression (95%CI: 0.205 − 0.986, *P* = 0.046).

### Splicing factor NOVA-1 accelerates the biogenesis of circBCAR3

To find out why circFAM13B is downregulated in patients with lymph node metastasis, we designed shRNAs targeting different splicing factors according to previous studies. We transfected shRNAs of QKI, ESRP, RBM NOVA and SRSF and detected the expression of circFAM13B in the constructed NPC stable lines (Supplementary Fig. 5A). The results showed that circFAMl3B but not the parental gene FAM13B expression decreased only after NOVA alternative splicing regulator 1(NOVA-1) knockdown, suggesting thatNOVA-1 is a key factor leading to circFAM13B formation (Supplementary Fig. 5B-C).

Therefore, this study systematically elucidated that the ER-associated circular RNA circFAM13B enhances the interaction between the RBM3 protein and the precursor uXBP1 mRNA of key signaling molecules in ER, promoting its degradation, resulting in reduced nuclear entry of the transcription factor sXBP1, and subsequently inhibiting the cytokine CST6 transcription, thus suppressing invasion, metastasis, and lymphatic vessel formation in NPC cells (Fig. 8H).

## Discussion

NPC is prone to lymphatic metastasis due to its well-developed lymphatic network. Approximately 70-80% of patients already have cervical lymph node metastasis at the time of initial diagnosis. A high incidence of early lymph node and distant metastases results in treatment failure in NPC patients [5]. Furthermore, lymph node metastasis affects the selection of treatment options and prognostic assessment. Therefore, our study aimed to uncover the molecular mechanisms of lymphatic metastasis in patients with NPC and explore potential molecular targets for predicting and targeting interventions against metastasis. This is crucial for improving the treatment outcomes of NPC. The motivation behind our study stems from the observation in clinical practice that even patients with the same primary nasopharyngeal tumor (T stage) exhibit significant heterogeneity in the extent of lymph node involvement (N stage). This prompted us to investigate the mechanisms underlying lymph node metastasis. circRNAs play various roles in tumor development and have the potential to serve as novel targets in cancer treatment. Multiple studies have shown the association of circular RNAs with lymphatic metastasis and angiogenesis [13–15]. To explore the mechanism of lymph node metastasis in NPC, we performed transcriptome sequencing of paired nasopharyngeal biopsy specimens from patients with T3/4N0 and T3/4N3 stages. We found that circFAM13B was downregulated in tumor tissues of patients with lymph node metastasis. CircFAM13B has been reported to play different tumor functions in bladder cancer and liver cancer, but it has not yet been reported in nasopharyngeal carcinoma [25, 26]. In vitro and in vivo functional experiments demonstrated that circFAM13B acts as a tumor suppressor in NPC, effectively inhibiting the invasion, metastasis, and lymphangiogenesis of NPC cells. We further explored how circFAM13B affects lymphatic metastasis in NPC and discovered that circFAM13B is localized to ER. It enhances the interaction between the downstream RNA-binding protein RBM3 and the key signaling molecule XBP1 in ER stress, leading to the degradation of uXBP1 mRNA, reduced nuclear translocation of sXBP1, and subsequently decreased transcriptional regulation of the CST6 gene. This results in the reduced secretion of CST6, which inhibits lymphangiogenesis and lymph node metastasis in NPC.

Currently, studies have explored lymph node metastasis in NPC. Zhou et al. observed that pigment epithelium-derived factor (PEDF) inhibited lymphatic vessel formation and tumor cell lymphatic metastasis in NPC by suppressing VEGF-C, a vascular endothelial growth factor [27]. Luo et al. found that Foxq1 induces angiogenesis through the EGFR signaling pathway, thereby promoting lymphangiogenesis and lymph node metastasis in NPC [28]. However, the molecular mechanisms underlying lymph node metastasis in NPC have not been fully elucidated and require further investigation. Apart from this, circRNAs play various physiological and pathological roles in the development and progression of tumors [9]. Several studies have demonstrated the involvement of circular RNAs in lymph node metastasis and angiogenesis [13, 14]. An et al. revealed that ubiquitination-mediated DDX39B-circNCOR1-SMAD7 signaling regulates lymph node metastasis in bladder cancer. Diao et al. revealed that extracellular vesicles containing circTLCD4-RWDD3 activated the transcription factor PROX1, a key regulator of lymphatic vessel formation, thereby promoting lymphatic metastasis in non-small cell lung cancer. Therefore, we investigated the mechanisms by which circular RNAs influence lymph node metastasis and angiogenesis in NPC.

Aberrant activation of the UPR sensors (IRE1α-XBP1, PERK-ATF4, ATF6) and their downstream signaling pathways have emerged as critical regulators of tumor angiogenesis, metastasis, and response to chemotherapy, targeted therapy, and immunotherapy. It has been reported that the PERK and ATF6 pathways of UPR are activated and promote cell survival and angiogenesis in endothelial cells in response to VEGF stimulation [29]. Additionally, in breast cancer, the IRE1α-XBP1 signaling pathway can activate the transcription of downstream Snail genes, enhancing the migratory and invasive capabilities of breast cancer cells [30]. Therefore, using FISH of circFAM13B, we observed its potential association with ER. Subsequently, we colocalized circFAM13B with ER using ER tracker and ER resident protein Erp72 plasmids, and successfully observed the enrichment of circFAM13B in ER using an ER isolation kit, indicating its presence on ER. We observed that circFAM13B overexpression leads to a decrease in the expression of the key downstream components of ER stress, uXBP1 (unspliced form) and sXBP1 (spliced form). Therefore, our primary focus was on two aspects of this mechanism: first, how circFAM13B regulates the expression of XBP1 and subsequently influences its nuclear translocation and regulation. Second, the effect of circFAM13B on lymphatic metastasis through its influence on the nuclear translocation of sXBP1.

To investigate how circFAM13B regulates XBP1 expression, we performed RNA pull-down and RIP assays to identify and validate the downstream RNA-binding protein of circFAM13B. We found that RBM3, a cold shock protein that plays important roles in various cellular processes, including neural repair, apoptosis, and damage, ER stress, and tumor development, is a potential interacting protein of circFAM13B [31–35]. Wellmann et al. discovered that RBM3 can inhibit the ER stress-induced PERK signaling pathway and prevent cell death by cooperating with NF90; however, its regulation of another pathway, XBP1, has not been reported. Therefore, we generated stable cell lines with RBM3 knockdown or overexpression and found that the expression of downstream uXBP1 and sXBP1 was affected, whereas the phosphorylation of IRE1α remained unchanged. This led us to speculate that RBM3 directly interacts with XBP1. To test this hypothesis, we performed RIP experiments using an anti-RBM3 antibody and found that RBM3 is directly bound to uXBP1 mRNA. However, the exact mechanism through which RBM3 regulates uXBP1 mRNA expression remains unclear. Xia et al. found that RBM3 influences the stability of downstream YAP-1 mRNA [21]. Bastide et al. demonstrated that the mRNA of ER membrane protein 3 enhanced translation initiation by binding to RBM3 [36]. Based on these findings, we hypothesized that RBM3 might also affect the stability of uXBP1 mRNA. Further experiments using actinomycin D showed that RBM3 overexpression promoted the degradation of uXBP1 mRNA. Moreover, when circFAM13B was overexpressed in RBM3-overexpressing cells, the stability of uXBP1 mRNA decreased further, indicating that circFAM13B enhanced the interaction between RBM3 and uXBP1, leading to its degradation. After XBP1 is spliced into sXBP1, it is translated into a transcription factor and translocated to the nucleus. To further elucidate the mechanism underlying the ER localization of circFAM13B, we found that RBM3, a circFAM13B-binding protein, is crucial for its ER localization. RBM3 localizes to the ER independently of circFAM13B. Importantly, RBM3 knockdown significantly reduced the co-localization of circFAM13B with the ER and abolished its enrichment in ER fractions. These results indicate that RBM3 is required for the ER localization of circFAM13B, while RBM3’s own ER localization is independent of circFAM13B. This observation is consistent with previous studies, including the research by Wellmann S et al. [35], who reported that RBM3 co-localizes with the ER protein PERK in the cytoplasm. These findings provide further insights into the role of RBM3 in the ER localization of circFAM13B and its subsequent effects on XBP1 mRNA stability and lymphatic metastasis in NPC.

In contrast, circFAM13B influences lymphatic metastasis by affecting the nuclear translocation of sXBP1. circFAM13B is mainly expressed in tumor cells, and previous studies have indicated that lymphatic metastasis is often associated with cytokines. For example, the upregulation of CCL2 induced by LNMAT1 can attract macrophages to the tumor, promoting lymphatic metastasis through VEGF-C secretion [37]. Therefore, we used a cytokine chip to sequence the CM of circFAM13B overexpression and knockdown stable cell lines. We identified three differentially expressed cytokines, including RANK, CEACAM-5, and CST6. Among these, CST6 showed the most significant difference. Furthermore, our transcriptome analysis revealed a significant downregulation of CST6. Subsequently, we validated this finding by ELISA of CST6 and identified CST6 as a potential target for further investigation. These findings suggest that circFAM13B exerts its inhibitory effect on lymphatic metastasis by regulating cytokine CST6.

The protein cystatin M/E encoded by CST6 has different functional effects in the progression of various tumors [38]. It has a tumor suppressor effect in non-small cell lung cancer [39], cervical cancer [40], prostate cancer [41], and gastric cancer [42], and is therefore considered a biomarker with prognostic significance. In studies on oral cancer [43], pancreatic cancer [44], thyroid cancer [45], and liver cancer [46], CST6 has been found to play a key role in tumor promotion. However, the expression of CST6 in NPC and its relationship with the prognosis of NPC patients are still unclear. Previous studies have shown that CST6 expression was associated with lymph node metastasis. In triple-negative breast cancer, CST6 expression is associated with a high risk of lymph node metastasis [47]. In colon cancer, CST6 expression in tumor tissues is significantly higher than that in normal colon tissues and is significantly associated with the vascular endothelial growth factor PDGFR and EMT signaling pathway [48]. The expression level of CST6 in the metastatic foci of squamous cell carcinoma of the oral cavity is 40 times that in the primary lesion [43]. Our study found that CST6 acts as a tumor-promoting molecule in NPC, promoting lymphatic endothelial cell tube formation and migration and that high expression of CST6 affects the prognosis of patients with NPC.

However, the relationship between XBP1 and CST6 remained unclear. Therefore, we conducted luciferase reporter experiments and found that overexpression of XBP1 activated the CST6 promoter. Furthermore, we performed ChIP truncation experiments and identified the − 900–600 bp region as the activation site for CST6, demonstrating that circFAM13B enhances the interaction between RBM3 and uXBP1, promoting its degradation and affecting the transcriptional regulation of CST6, thereby influencing lymphatic vessel formation.

In this study, the popliteal lymph node metastasis model used, in which NPC cells are injected into the mouse footpad, can be used to sensitively quantify lymph node metastasis in vivo, but it also has several limitations. First, the microenvironment of the footpad is very different from that of the nasopharynx. Second, the interstitial fluid pressure (IFP) within the tumor is usually much higher than the fluid pressure in the surrounding host tissue, which affects the different lymphatic fluid return and filtration rates, resulting in some differences in lymph node metastasis results [49]. Finally, the BALB/c nude mouse model lacks effective adaptive immunity, which is often used to test drug treatment studies of human tumor xenografts [50].

## Conclusion

In conclusion, this study systematically elucidated the molecular mechanism by which circFAM13B inhibits lymphatic metastasis by affecting ER stress. We found that circFAM13B functions as a tumor suppressor gene in NPC and is localized to ER. It enhances the interaction between downstream RBM3 and the key signaling molecule precursor uXBP1 mRNA, promoting its degradation and resulting in reduced nuclear translocation of the transcription factor sXBP1. This inhibition of the transcriptional regulation of CST6 leads to decreased cytokine CST6 secretion, thereby suppressing invasion, metastasis, and lymphatic vessel formation of NPC cells.

## Methods and materials

### Cell culture

Keratinocyte serum-free medium (Invitrogen, USA) supplemented with bovine pituitary extract (BD Biosciences, USA) was used to grow the NPEC line NP69. Human NPC cell lines (HK1, CNE2) were maintained in RPMI 1640 (Invitrogen) supplemented with 5% fetal bovine serum (FBS, Gibco, USA). All NPEC and NPC cell lines were generously provided by Professor Musheng Zeng (Sun Yat-sen University Cancer Center, China). 293FT cells obtained from ATCC were cultured in Dulbecco’s modified Eagle’s medium (Invitrogen) supplemented with 10% FBS.

### Fluorescence in situ hybridization (FISH)

Cy3-labeled specific fluorescence probes were designed according to the fusion site of the circular RNA. NPC cells were spread onto glass-bottom dishes, fixed with 4% paraformaldehyde at room temperature (RT) for 15 min, and permeabilized on ice for 5 min. The probes and pre-hybridization solutions were mixed to prepare the hybridization solution, which was dropped onto the cells to cover them. The dishes were incubated overnight at 37 °C. Excess unbound probes were washed off the following day. DAPI was used to stain the nucleus, and fluorescence microscopy or confocal microscopy was used to observe and capture images.

### Construction of stable cell lines

Plasmids containing shRNA targeting the fusion site of circFAM13B or overexpressing circFAM13B were constructed and verified for the correct sequences by sequencing. 293T cells were transfected and when the cells reached a 70% fusion rate, they were co-transfected with the packaging plasmids. Transfection was performed using Lipofectamine 3000 (Invitrogen). The viral supernatant was collected 48 h after transfection, filtered, and used to infect the target cells. Puromycin was added to the culture medium for 7 days to select stable knockdown or overexpression cell lines, and qRT-PCR was used to verify the expression of the target gene circFAM13B in the stable cell lines.

### Overexpression plasmids construction

The overexpression vector used was the pLC5-ciR from GeneSE Biotech Co., Ltd. (Guangzhou, China). Initially, EcoRI and BamHI double digestion was performed to remove the Stuffer in the middle of the vector. Subsequently, the empty vector was recovered and connected with the target circRNA. The target circRNA was amplified according to the design of PCR primers. The primers for CircFAM13B are as follows:

Primer-F: 5’CGGAATTCTAATACTTTCAGAATGAAGAAAATACCCAGCACCC 3’.

Primer-R: 5’CGGGATCCAGTTGTTCTTACACACACCACACTTTGCTGTTGTAAA 3’.

After successful construction, sequencing was performed using cloning primers.

### Gene knockdown by sirna/shrna

Small interfering RNAs were designed and synthesized by RiboBio (Guangzhou, China). The siRNA sequences are shown in Supplementary Table 4. For siRNA transfection, Lipofectamine^®^3000 was used as transfection agent according to the user protocol. Sh-circFAM13B lentivirus were generated by GeneCopoeia (USA) using psi-LVRU6H lentiviral vector based on the si-circFAM13B sequence.

### RNA-seq

RNA quality was assessed using 1% agarose gels, NanoPhotometer^®^, and the RNA Nano 6000 Assay Kit on the Bioanalyzer 2100 system. For library preparation, 5 µg of RNA per sample was processed: ribosomal RNA was removed, linear RNA was digested with RNase R, and libraries were generated using the NEBNext^®^ Ultra™ Directional RNA Library Prep Kit. Sequencing on an Illumina platform produced 150 bp paired-end reads. Raw fastq data were cleaned using in-house Perl scripts. Reads were aligned to the reference genome using Bowtie2 (v2.2.8), and circRNAs were identified with find_circ and CIRI2. Gene expression was quantified by TPM normalization, and differential expression was analyzed using DESeq for samples with replicates and DEGseq with edgeR for those without. These methods ensured accurate and reliable RNA-seq analysis.

### ChIP analysis

The ChIP experiments were performed using the EZ-Magna ChIP A/G kit (Millipore, MA, USA). A total of 1 × 10^7^ cells were fixed in 1% formaldehyde at room temperature for 10 min, and the nuclei were isolated with nuclear lysis buffer supplemented with a protease inhibitor. The chromatin DNA was sonicated and sheared to lengths between 100 and 200 bp. The sheared chromatin was immunoprecipitated at 4 °C overnight using an anti-XBP1 antibody (Abcam, MA, USA). Normal mouse IgG was used as the NC, and an anti-RNA pol II antibody (Mil-lipore) was used as the positive control. The ChIP-qPCR primers are listed in Supplementary Table 4.

### RNA pull-down and RNA immunoprecipitation (RIP)

This study utilized the RNA pull-down kit (BersinBio™, Guangzhou, China), with the target RNA probe and NC probe designed by RiboBio (Guangzhou, China), and the silver staining kit was the fast silver staining kit (Beyotime, Shanghai, China). For the RNA pull-down, biotin-labeled target RNA and NC probes were mixed in 30 µl RNase-free water at a 1 µg/1000nt ratio, then denatured at 90 ℃ for 2 min and cooled on ice for 2 min. Next, 25 µl DEPC water and 40 µl RNA structure buffer was added, mixed, and allowed to stand at room temperature for 20 min. Meanwhile, 80 µl magnetic beads were washed with TES, and 40 µl magnetic beads were added to RNase-free EP tubes along with 100 µl DEPC water, 100 µl RNA probe, and 200 µl TES. After mixing, samples were rotated at room temperature for 0.5 h, supernatant was removed with a magnetic rack, and beads were washed twice with 1 mL TES for 1 min each. Finally, 2 × 10^7 cells were collected, 1.7 mL RIP buffer and 17 µl protease inhibitors were added, and the mixture was placed on ice for 10 min. After mixing, the sample was frozen at -80 ℃ for 10 min and thawed on ice. Cells were centrifuged at 15,000 g for 15 min at 4 ℃, and the supernatant was transferred to an RNase-free tube. Cells were ultrasonically disrupted for 3 min (35% power, 2 s on, 5 s off). Post-ultrasonication, 25 µl DNase was added to remove nucleic acids and incubated for 1 h. Then, 60 µl Agarose beads and 16 µl DTT were added, mixed, and rotated at 4 ℃ for 30 min. The sample was centrifuged at 4000 g for 30 s, and 100 µl supernatant was taken as input, with the rest divided into experimental and control groups. The probe-magnetic bead complex was mixed with the cell extract, rotated for 2 h, and the beads were collected, washed six times with NT2 buffer. Finally, 50 µL 2×simple buffer was added and heated at 100 ℃ for 5 min. Finally, 15 µl was taken for silver staining, 20 µl for LC-MS detection, and the remaining 15 µl could be used for PAGE-western blot verification after the mass spectrometry results were obtained. The uncropped gels are summarized in Supplementary Figure.

The RIP assay was performed using the EZ-Magna RIP kit (Millipore, MA, USA). Briefly, 1 × 10^7^ cells were harvested and lysed with RIP lysis buffer with one freeze–thaw cycle. Cell extracts were co-immunoprecipitated using anti-RBM3 (Abcam, MA, USA), and the retrieved RNA was subjected to qRT-PCR analysis. Normal mouse IgG was used as the NC. For qRT-PCR analysis, U1 RNA was used as a non-specific control.

### Real-time qPCR

Total RNA was extracted using TRIZOL Reagent (Thermo Fisher Scientific) and reverse-transcribed into cDNA using a Reverse Transcription Kit (Bio-Rad), following the manufacturer’s protocol. All qPCRs were performed using the SYBR Green Master Mix (Applied Biosystems). mRNA expression was quantified using the comparative Ct method. GAPDH was used as an internal control.

### RNA interference

NPC cells were transiently transfected using Lipofectamine RNAi MAX (Invitrogen). CNE2 or HK-1 cells were seeded at 2 × 10^5^ cells/well in 6-well plates. After 12 h, cells were transfected with 40 pmol of siRNA purchased from Ruibo (Guangzhou, China). Effective silencing was achieved–48–72 h after transfection. The siRNAs used are listed in the Supplementary Table.

### CCK‑8 and colony formation assays

A Cell Counting Kit-8 (CCK-8, #CK04, Dojindo, Tokyo, Japan) was utilized to assess cell viability. The CCK-8 reagent (10%) was diluted to the working solution and added to a 96-well plate followed by incubation at 37 °C for 2 h. Optical density (OD) values at wavelength of 450 nm were assessed using a microplate reader (#E0226, Beyotime).

For colony formation assay, HK1 and CNE2 cells were seeded into six-well plates with 600 cells per well. On the second week after culture, cells were fixed with para-formaldehyde (#P0099, Beyotime) and stained by 0.1% crystal violet (#C0121, Beyotime).

### Wound healing assay

A monolayer of nasopharyngeal carcinoma cells was inoculated into a 6-well plate, scratched with a sterile 200 µL pipette tip, and cultured in serum-free medium. After 0 and 24 h of culture, photos were taken and the scratch width was measured.

### Transwell assays

After transfection, cells (In order to more intuitively demonstrate the differences between the experimental group and the control group, different inoculation densities were used in different control groups) were seeded into pre-equilibrated 8 μm pore Transwells (3374, Corning, USA) inserts coated with Matrigel (for invasion) or without Matrigel (#356237, BD Biosciences, USA) (for migration). After 24 h of incubation, cells in the upper chamber were removed with a cotton swab, and cells in the lower chamber were fixed in 2% paraformaldehyde for 10 min and stained with crystal violet. The number of migrated or invaded cells was counted in six randomly selected fields under a microscope (ECLIPSE Ti, Nikon, Japan).

### Cytokine microarray analysis

Cells were seeded in 6-well plates and cultured overnight. The medium was changed to serum-free RPMI-1640 after three washes with sterile PBS. After 48 h, the supernatants were harvested and centrifuged at 3,000 rpm for 5 min. Human cytokine antibody array GS440 and human chemokine array C1 (AAH-CHE-1) from Raybiotech, Inc. (Norcross, Georgia, USA) were used to measure soluble cytokines. The microarray assay was based on sandwich ELISA with two sets of anti-cytokine antibodies. Cytokines in all samples were measured in triplicate. After incubation with the antibody cocktail and dye, plates were washed five times with gentle shaking. The hybridized arrays were scanned for fluorescence using a laser scanner at a Cy3 wavelength. Data were extracted using microarray analysis software (GenePix, ScanArray Express).

### Western blotting

Western blotting (WB) was performed on cell lysates prepared in RIPA buffer supplemented with a protease-phosphatase mix (Pierce). Briefly, the cell lysate was separated on a Tris-glycine sodium dodecyl sulfate-polyacrylamide gel and transferred onto a PVDF membrane. Membranes were blocked in 5% non-fat milk prepared in TBS supplemented with 0.1% TWEEN-20 (TBST) for 1 h at RT followed by overnight incubation at 4 °C with primary antibodies, which were diluted in 1:1,000. Secondary antibodies were used at a concentration of 1:3,000 for 1 h at RT. GAPDH or α-tubulin served as internal control. Bands were quantified using Image J, and normalized to loading controls.

### Naked mouse tail vein injection in lung metastasis experiment and footpad injection in popliteal lymph node metastasis experiment

Female nude mice aged 4–6 weeks were quarantined and acclimatized. For tail vein injection, a cell concentration of 1 × 10^6^/150 µL was injected into the tail vein and the growth condition of the nude mice was observed every other day; after a specific period, the mice were euthanized by cervical dislocation, and lung tissue was dissected out, fixed with formalin-acetic acid-alcohol and observed for tumor metastasis. In addition, some tissues were fixed with formalin, embedded in paraffin, sliced, and stained with HE, and the differences between groups were statistically analyzed. For footpad injection, a cell concentration of 5 × 10^5^/25 µL was injected into the footpad of the mice, and the mice’s vitality was observed every other day. After a period, the footpad was observed for tumor formation, and the mice received an intraperitoneal injection of a fluorescent substrate to observe fluorescent signals in both the footpad and popliteal lymph nodes. After the fluorescent signal was detected, the mice were euthanized by cervical dislocation, and the footpad tumor tissue and popliteal lymph nodes were dissected to observe the size of the lymph nodes in the control and experimental groups. The lymph nodes were fixed with formalin, embedded in paraffin blocks, sliced, and subjected to HE and immunohistochemical staining to confirm the migration of tumor cells and perform intergroup data analysis. The parental nasopharyngeal carcinoma cell line used in the in vivo experiments of this study was HK1.

### Actinomycin D test

Nasopharyngeal carcinoma cells were inoculated in 6-well plates. When the cell density grew to 60%, actinomycin D (the concentration was selected according to the preliminary experiment, and the initial concentration was 5 µg/mL) was added. Cells were collected at different time periods (selected according to the preliminary experiment results of different cell lines), washed twice with PBS, and then 0.5 ml trizol was added to mix. The suspension was transferred to a nuclease-free EP tube and stored in a -80 ℃ refrigerator. After all cells were collected at all time periods, RNA was extracted and reverse transcribed, and then fluorescent quantitative PCR was performed to detect the relative expression level of XBP1 mRNA.

### Enzyme-linked immunosorbent assay (ELISA)

We used the kit of Signal way Antibody Company (Maryland, USA) to detect CST6 in cell culture supernatant and patient serum. Before the experiment, the reagents and samples of the kit need to be rewarmed at room temperature for half an hour, and the standard is diluted at the same time, and a continuous concentration gradient is configured: 0.156 ng/ml, 0.312 ng/ml, 0.625 ng/ml, 1.25 ng/ml, 3,5 ng/ml, 5ng /ml, 10 ng/ml and a blank control well is set. Add 100 µl of standard and cell supernatant (patient serum) to each well, seal it with a plate cover, and place it in a 37 ℃ incubator for incubation for 1.5 h. After the incubation is completed, use a multi-channel automatic washing machine to wash the plate, add washing solution to the automatic washing machine, let it stand for 1 min, and repeat the washing 4–6 times to ensure thorough cleaning. Then add 100 µl CST6 antibody to each well, seal with a plate cover, incubate in a 37 ℃ incubator for 1.5 h, and repeat washing 4 times with a washing machine. Then add 90 µl substrate solution to each well, continue to seal and incubate at 37 ℃ for 20 min, avoid light during incubation, incubation time should not exceed half an hour, and the solution will be blue after the incubation. Finally, add 50 µl stop solution, the solution will turn yellow after addition, and shake to mix. Immediately run the microplate reader and measure at 450 nm. Compare the measured sample absorbance value with the standard absorbance value, draw a standard curve, and calculate the sample concentration.

### Lymphangiogenesis experiment

Stable NPC cell lines were cultured until they reached 70% confluence and then transferred to an FBS-free medium for 24 h to obtain a CM. Lymphatic endothelial cells were normally cultured, digested, and counted, and the Matrigel matrix gel was added to the wells of a 96-well plate. The gel was allowed to solidify, and lymphatic endothelial cells were added on top of the gel and cultured in the CM. Tube formation was observed under a microscope after 24 h. The tube branch length was measured by software ImageJ (National Institutes of Health, USA) with the “Angiogenesis Analyzer” plugin. Each experiment was repeated at least three times.

### Migration assay

The migration assay of HLECs was performed in 8 μm 24-well Boyden chambers (Corning). HLECS (4*10^4^) were suspended in 100 µl RPMI 1640 medium and added to the upper chambers, and then, 600 µL RPMI 1640 medium (including 10% FBS), with or without CNE2-CM, recombinant CST6 (40 ng/mL) added in the lower chamber. The cells were allowed to migrate for 24 h and then fixed for microscopy-based observation. The mean number of cells in five microscopic fields was calculated.

### Dual luciferase reporter gene assay

The dual luciferase reporter gene assay used a kit from Promega (Madison, USA), and also purchased CST6 promoter plasmid (FR01-CST6) and XBP1 promoter plasmid (FR01-XBP1) from Hong Xun Biotechnology(Suzhou, China). First, nasopharyngeal carcinoma cell lines were used for transfection. Nasopharyngeal carcinoma cells were plated in 6-well plates to a cell density of 70%. 3.75 µl lipo3000 reagent was first diluted with 100 µl preheated Opti-MEM medium, and then mixed plasmids (2.5 ng) were diluted with 100 µl preheated Opti-MEM medium. The mixture was mixed evenly, and then mixed and incubated for 20 min. The mixed reagent was added to 293T cells and placed in a cell culture incubator for culture. After 36–48 h, 200 µl of supernatant was collected. The GLuc working solution was placed at room temperature and protected from light for 30 min. Add 100 µl GLuc working solution to each well of the 96-well plate, then continue to add 10 µl supernatant, shake and mix, incubate at room temperature for 60 s, and read the fluorescence signal intensity value of Gluc in the microplate reader. Next, place 200 µl cells in a 65 ℃ water bath for 15 min, and then place on ice. Prepare 1×AP buffer working solution and 1×SEAP working solution in advance, and incubate the prepared working solution at room temperature in the dark for 20 min. Add 100 µl SEAP working solution to each well of the 96-well plate, then continue to add 10 µl supernatant, shake and mix, incubate at room temperature for 15 min, and read the fluorescence signal intensity value of SEAP in the microplate reader. Calculate the activity of the CST6 promoter reporter gene based on the ratio of Gluc/SEAP.

### Immunohistochemistry (IHC) and Immunofluorescence (IF)

Formalin-fixed, paraffin-embedded tissue specimens were prepared and subjected to deparaffinization, rehydration, antigen retrieval, and endogenous peroxidase inactivation. Antigen retrieval was performed using a pressure cooker for 3 min in 0.01 M citrate buffer (pH 6.0). The slides were incubated with primary antibodies against XBP1 and pan-CK respectively at 4 °C overnight. After washing, the slides were incubated with rabbit or mouse horseradish peroxidase-conjugated secondary antibodies and visualized.

Cells to be used for immunofluorescence were cultured in glass-bottom dishes. At the time of harvesting, the cells were fixed with 4% paraformaldehyde, permeabilized, and blocked with PBS containing 5% BSA and 0.3% Triton X-100. Subsequently, cells were incubated overnight with primary antibodies at 4 °C, followed by incubation with Alex Fluor 594-conjugated anti-rabbit IgG. The cells were then stained with DAPI to visualize nuclear DNA. A Zeiss confocals microscope (LSM880, Germany) was used to visualize the images.

### Flow cytometry

Intracellular flow cytometric analysis was performed on HK1 and CNE2 cells that were fixed with 4% paraformaldehyde and permeabilized with 90% methanol. Cells were harvested and washed with PBS, then cells were resuspended in 100 µL PBS containing 10^6^ cells. Antibodies against XBP1 were added and the cells were incubated for 30 min in the dark. The cells were then washed twice by centrifuge and resuspended in PBS. Flow cytometry was performed using the FACS Calibur.

### RNA scope

This experiment uses Advanced Cell Diagnostics’ BaseScope™ Red Kit (California, USA). First, place the experimental reagent AMP1-8 at room temperature to reheat, and heat the probe in a 40 ℃ water bath for 10 min for use. After the probe cools to room temperature, add 4 drops to the tissue chip and place it in a hybridization oven at 40 ℃ for 2 h. After hybridization, wash it twice with PBST for 2 min each time. Then, add BaseScope™ v2 AMP1-8 in sequence according to the steps of the hybridization probe. After AMP8 is incubated, add 120 µl RED working solution. After incubation at room temperature for 10 min, wash the slide, stain it with 50% hematoxylin for 2 min, and quickly wash the tissue chip in clean water for 2 min. Finally, soak it in 0.02% ammonia water for 30 s, wash the tissue chip with PBST, place it in a 60 ℃ oven for 15 min, seal it with a quenched sealing agent, scan it with a Jiangfeng scanner, and use HALO software for quantitative analysis.

### Clinical sample collection and storage

Clinical tissue samples were obtained post-nasopharyngeal biopsy, fixed in 10% formalin, and processed through dehydration, clarification, and paraffin embedding. Sections were cut, mounted, and dried at 37 °C, then stored at room temperature or long-term at -80 °C. Serum samples were collected via venipuncture, allowed to clot, and then centrifuged at 4 °C. The serum was aliquoted and stored at -80 °C until analysis. All procedures followed ethical standards with informed consent, approved by Sun Yat-sen University Cancer Center, with complete clinical data.

### Statistical analysis

All quantitative data are presented as the mean ± standard deviation from at least three independent experiments. All experimental data were analyzed and graphed using GraphPad Prism software (version 10.0). The chi-square test (χ2 test) for non-parametric variables and Student’s t-test or one-way analysis of variance (ANOVA) for parametric variables (two-tailed tests) were used to identify statistically significant data. OS and DFS were evaluated using the Kaplan–Meier method, and the log-rank test was used to compare differences between groups. A multivariable Cox proportional hazards model was used to estimate independent prognostic factors. Bioinformatics analysis and statistical calculations were performed using SPSS 23.0 and R 3.6.3 software. The GSEA analysis was conducted utilizing the MSigDB database, which can be accessed at http://www.gsea-msigdb.org/gsea/msigdb/index.jsp. Notably, the significantly enriched gene sets were identified with a threshold of|NES| greater than 1, a nominal (NOM) p-value less than 0.05, and a false discovery rate (FDR) value less than 0.25. For the enrichment analysis, we employed GSEA version 4.3.0 software. *p* < 0.05 was considered statistically significant; *p* > 0.05, marked as ns; *p* < 0.05, marked as *; *p* < 0.01, marked as **; and *p* < 0.001, marked as ***.

## Electronic supplementary material

Below is the link to the electronic supplementary material.

Supplementary Material 2

Supplementary Material 1

## Data Availability

No datasets were generated or analysed during the current study.
