# Supplementary material for: Endoplasmic reticulum-localized circular RNA FAM13B restrains nasopharyngeal carcinoma lymphatic metastasis through downregulating XBP1
Source: J Exp Clin Cancer Res. 2025 Jul 31;44:223. doi: 10.1186/s13046-025-03468-7 (PMC12312493; doi:10.1186/s13046-025-03468-7)
Supplement: Supplementary file 1 — Supplementary Material 2 [file 13046_2025_3468_MOESM1_ESM.pdf]

Figure1F

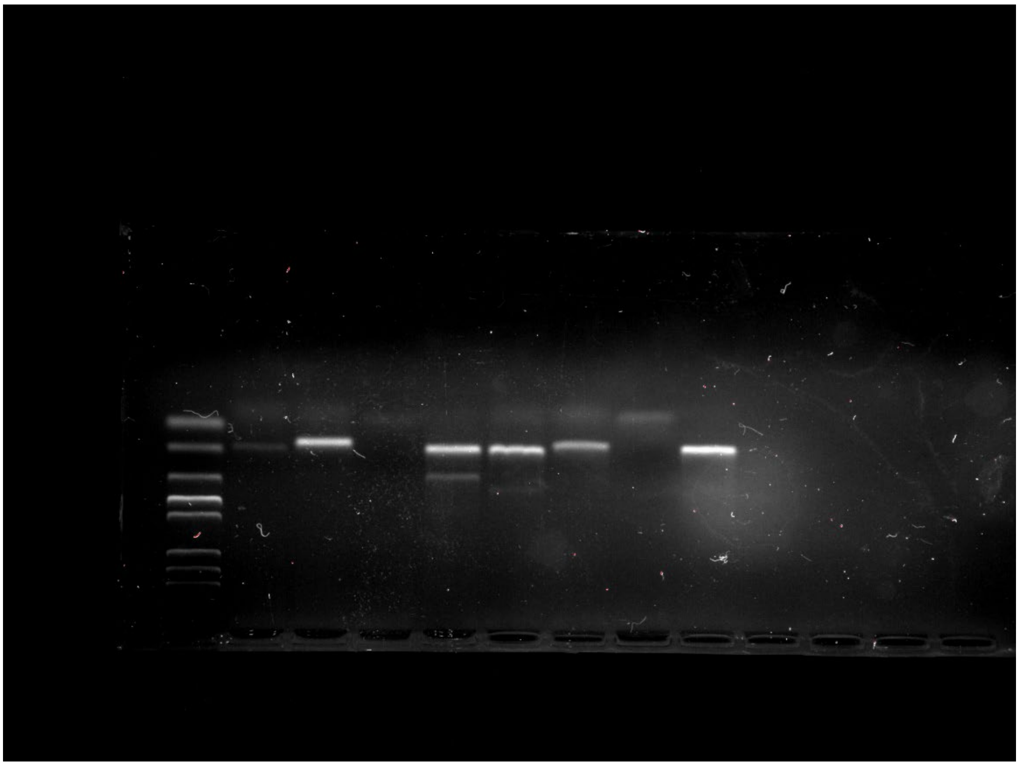

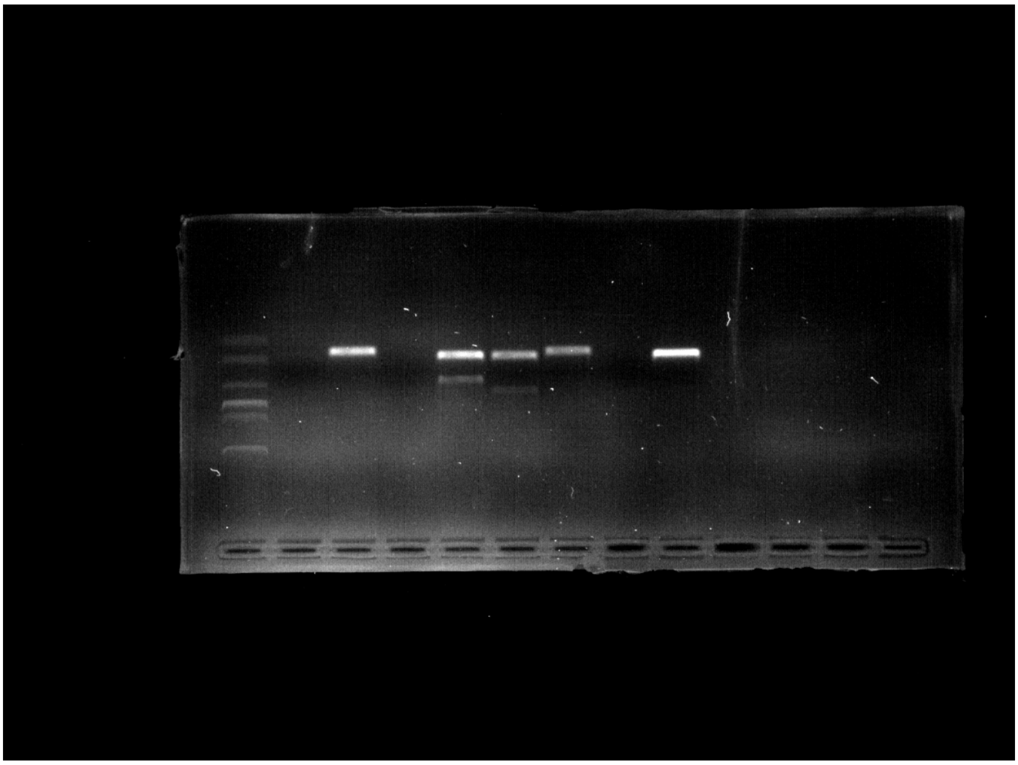

Figure4E

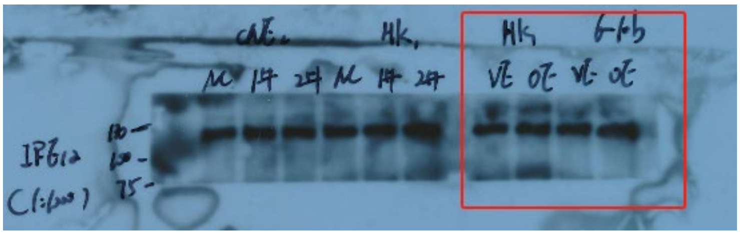
IRE1a

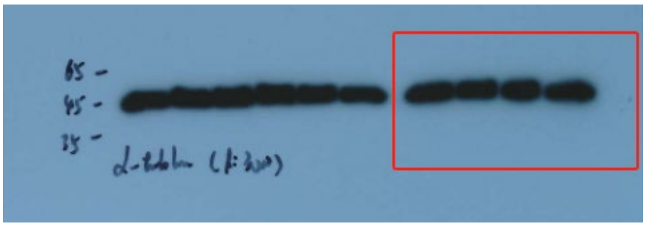
IRE1a-tubulin

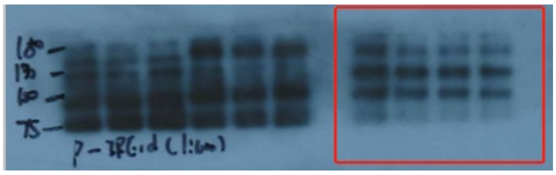
P-IRE1a

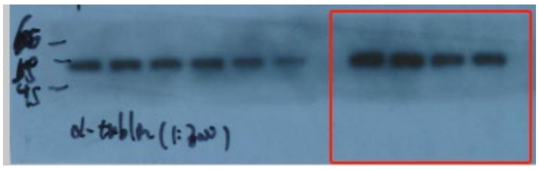
P-IRE1a-tubulin

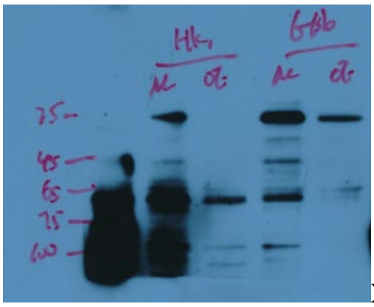
XBP1

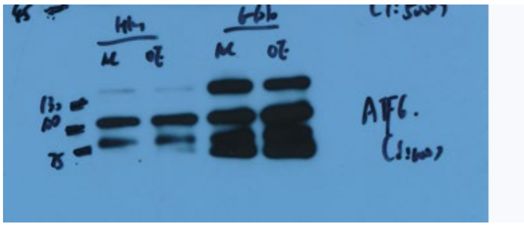
ATF6

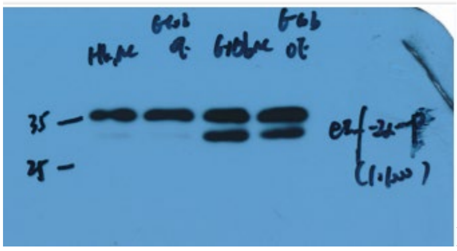
P-EIF2a

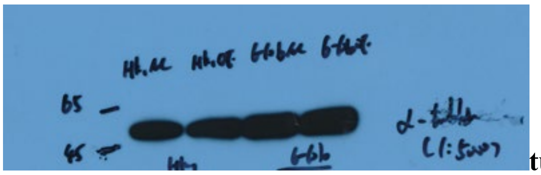
tubulin(XBP1-ATF6-P-EIF2a)

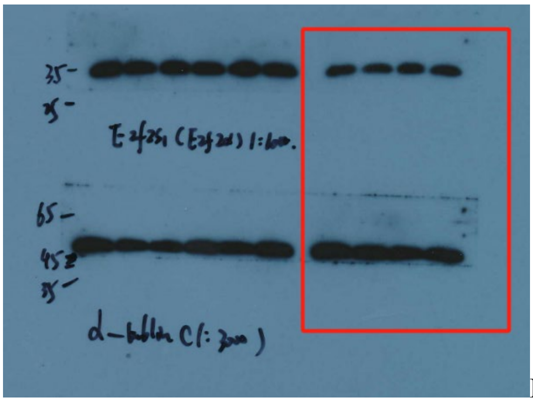
EIF2a-tubulin

Figure4F

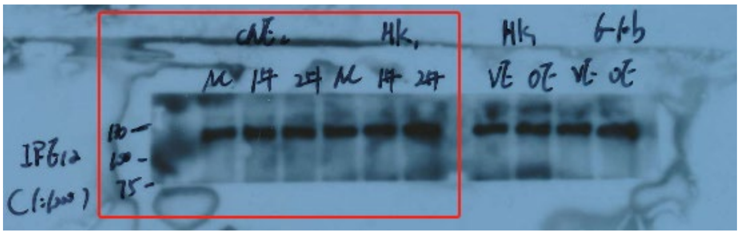
IRE1a

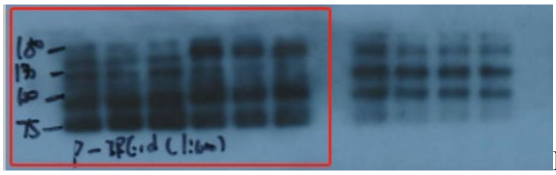
P- IRE1a

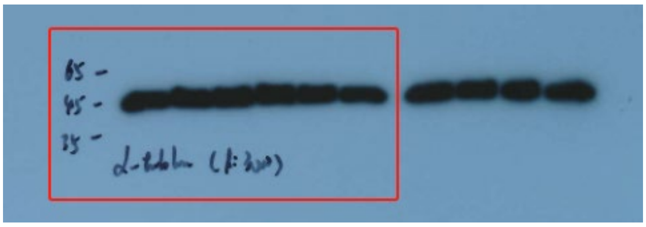
IRE1a-tubulin

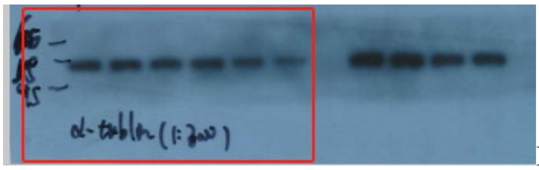
P- IRE1a-tubulin

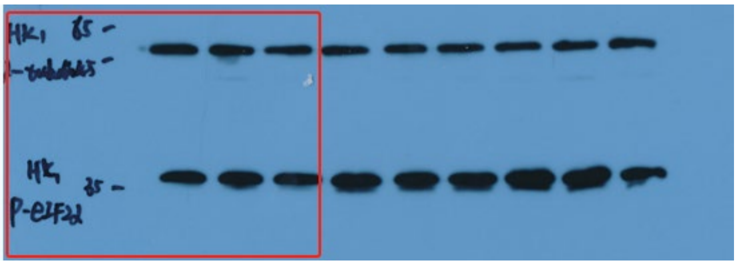
HK1-eif2a+tubulin

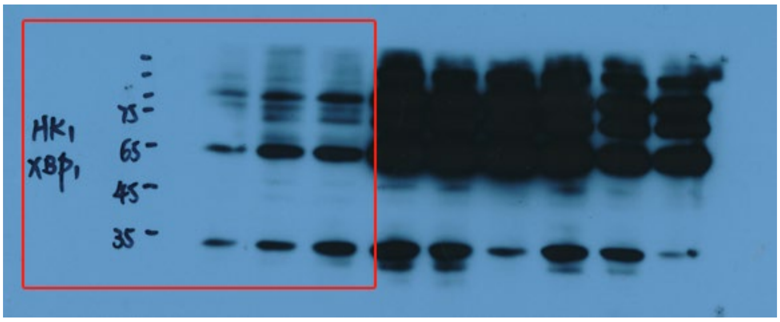

HK1-XBP1

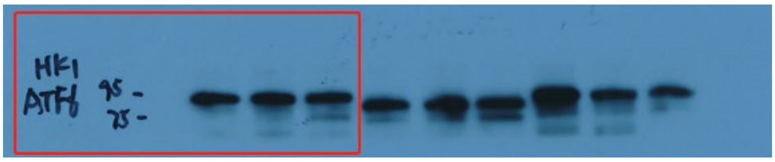

HK1-ATF6

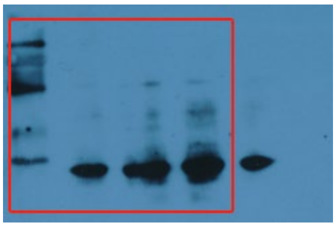

CNE2-XBP1

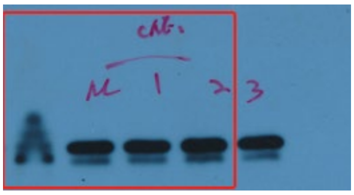

CNE2-ATF6

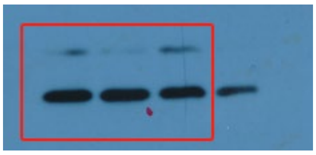

CNE2-eif2a

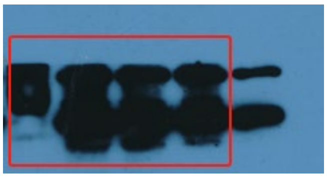

CNE2-tublin

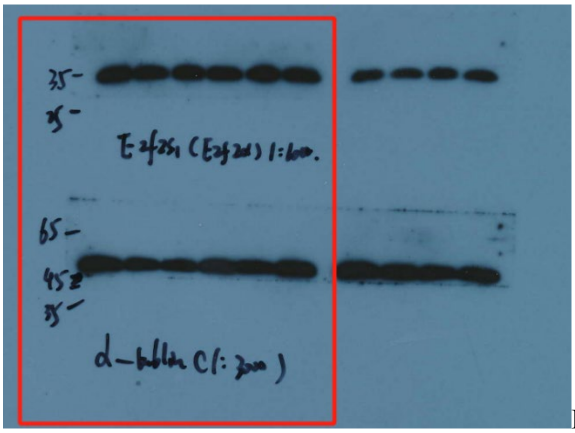

EIF2a

Figure5A

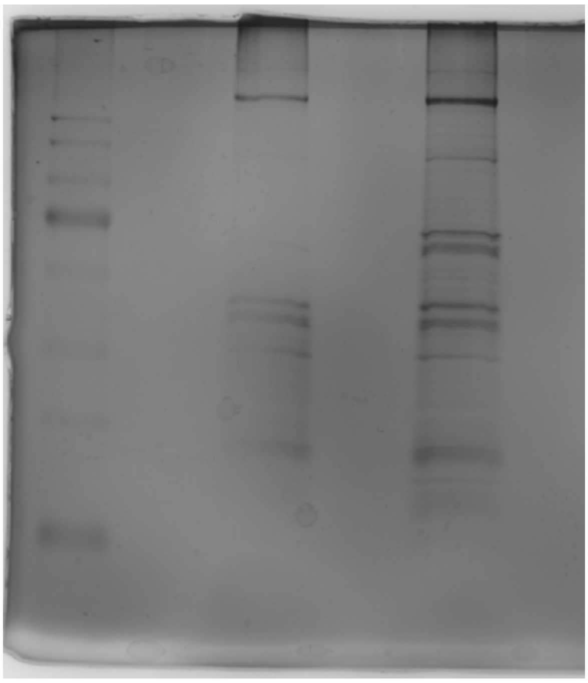

Figure5D

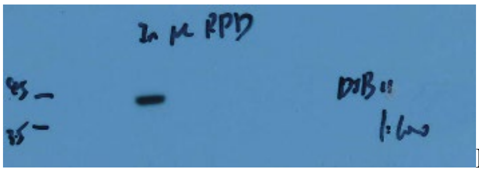

DJB11

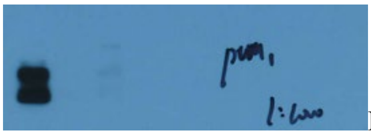

PUM1

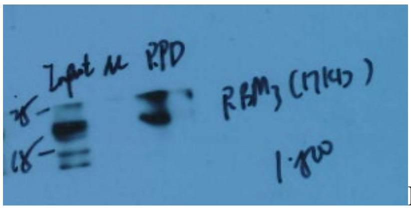

RBM3-1

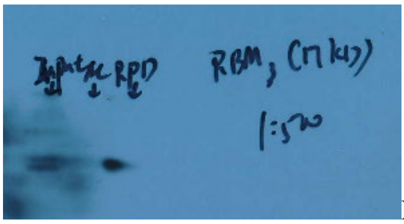

RBM3-2

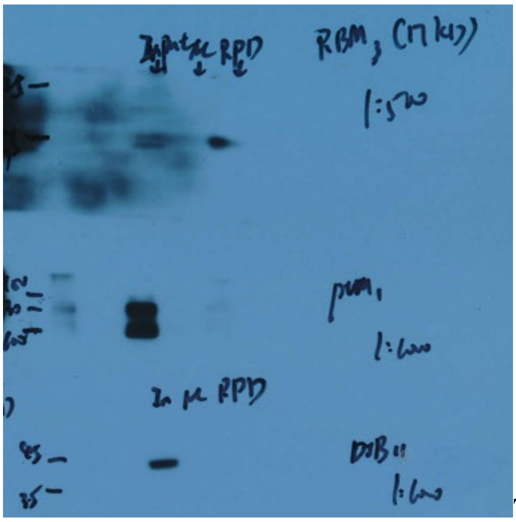

TOTAL

Figure5F

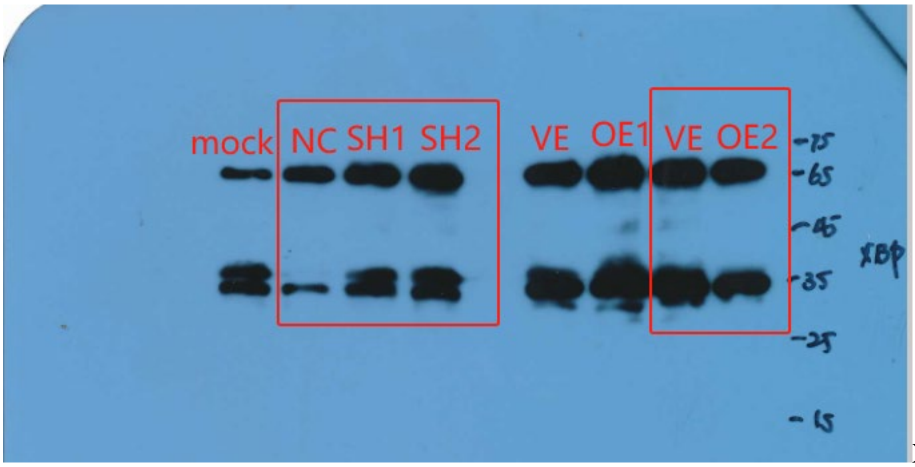

XBP1

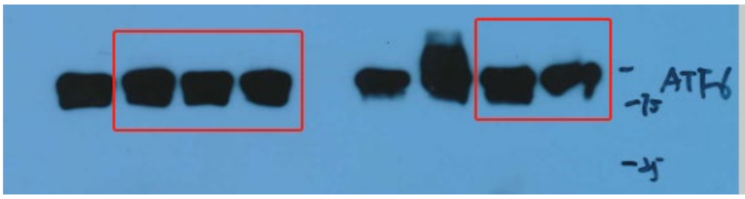

ATF6

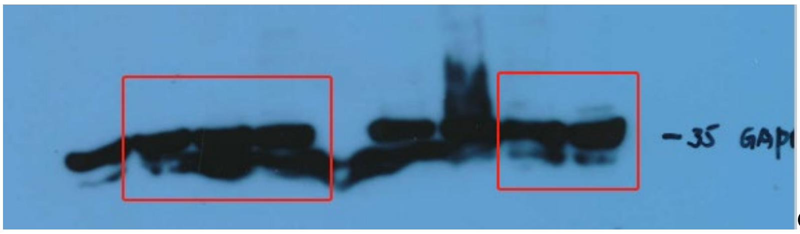

GAPDH

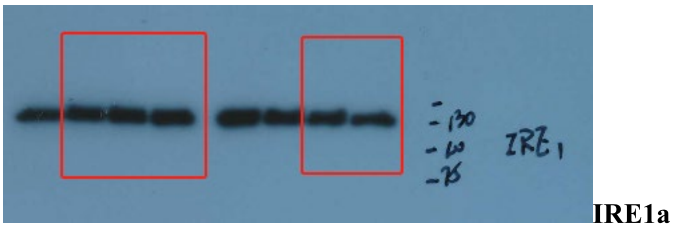

IRE1a

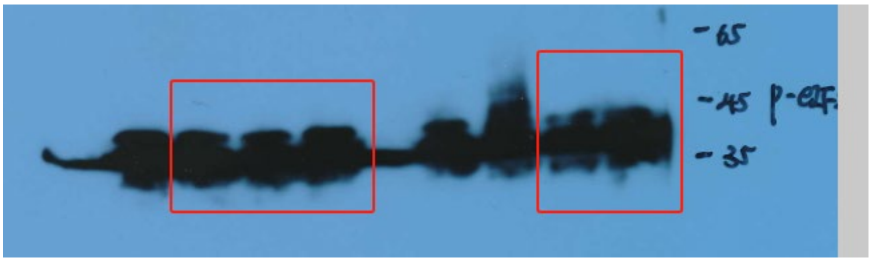

P-eif2a

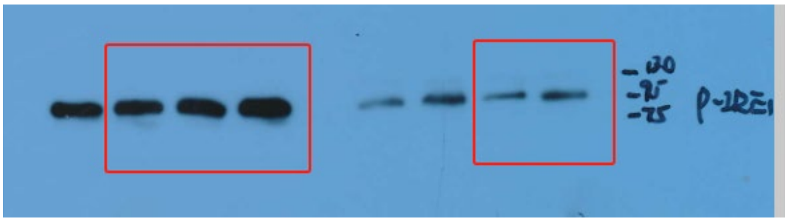

P-IRE1a

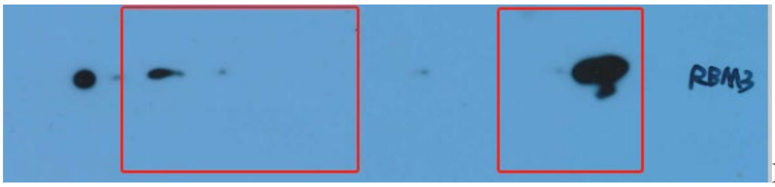

RBM3

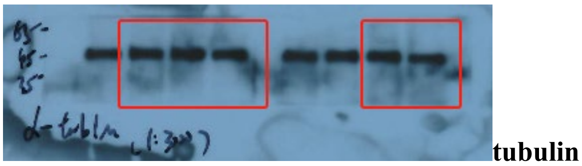

tubulin

Sig1

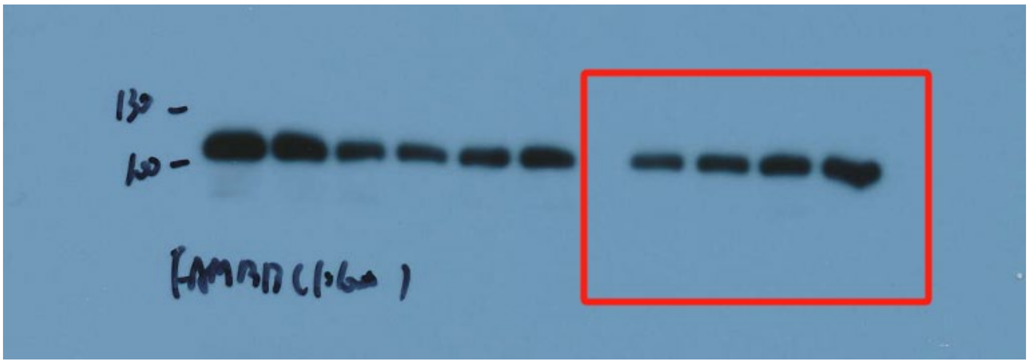

Sig1B-FAM13B-OE

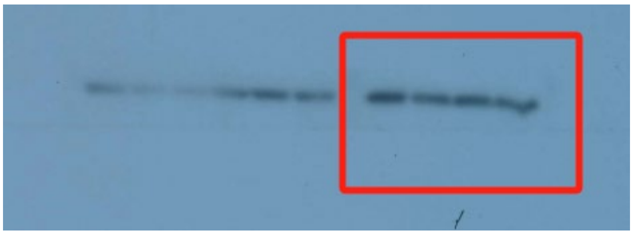

Sig1B-tubulin-OE

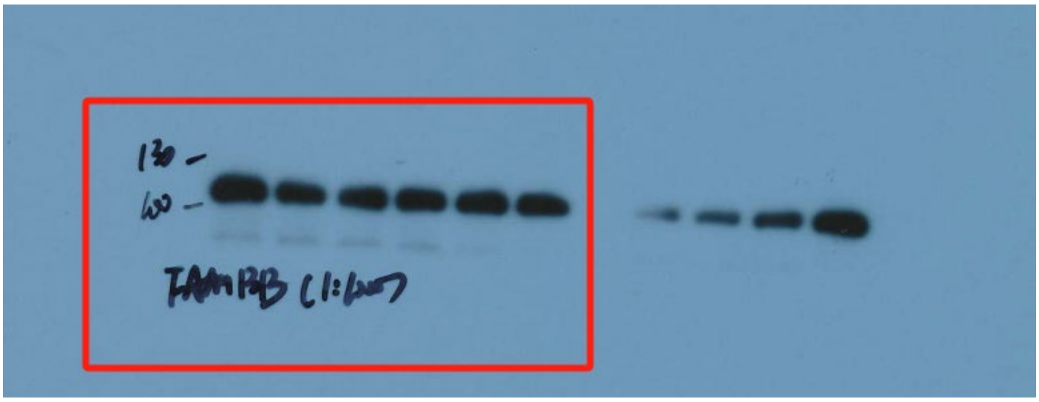

Sig1B-FAM13B-SH

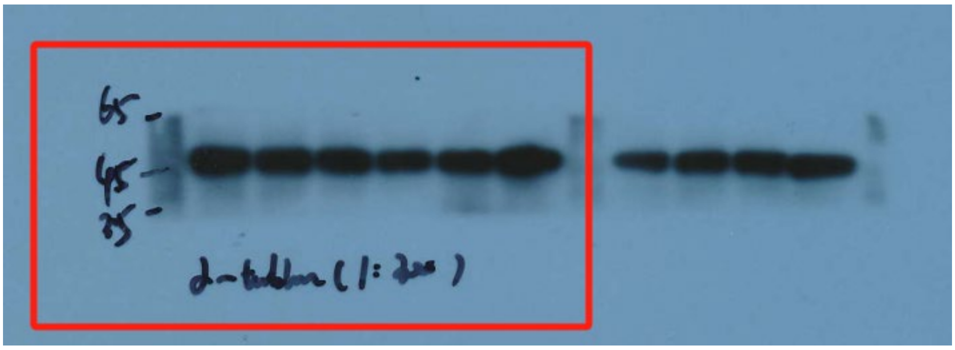

Sig1B-tubulin-SH
