# Supplementary material for: Endoplasmic reticulum-localized circular RNA FAM13B restrains nasopharyngeal carcinoma lymphatic metastasis through downregulating XBP1
Source: J Exp Clin Cancer Res. 2025 Jul 31;44:223. doi: 10.1186/s13046-025-03468-7 (PMC12312493; doi:10.1186/s13046-025-03468-7)
Supplement: Supplementary file 2 — Supplementary Material 1 [file 13046_2025_3468_MOESM2_ESM.docx]

**Supplementary Information**

Supplementary Figure 1: CircFAM13B does not affect the proliferation of NPC cells. A. qPCR of circFAM13B expression in overexpression and knockdown stable cell lines. B. Immunoblotting of FAM13B in circFAM13B overexpression and knockdown stable cells. C. CCK8 proliferation curve of stable circFAM13B overexpression cells; D. CCK8 assay of circFAM13B knockdown stable cells; E-F. Colony formation assay of circFAM13B overexpression and knockdown stable cells. The error bars represent standard deviations of three independent experiments. **p*<0.05, ***p*<0.01 and ****p*<0.001.

Supplementary Figure 2: The expression of circFAM13B is negatively correlated with XBP1 in NPC patients. A. Representative images of circFAM13B RNA SCOPE and XBP1 immunohistochemical staining in normal and NPC patients. Scale bar: 100µm (applies to all images); B. Spearman correlation analysis of circFAM13B and XBP1 expression. **p*<0.05, ***p*<0.01 and ****p*<0.001.

Supplementary Figure 3. CircFAM13B does not affect the ER location of RMB3 in NPC cells. A. RBM3 immunofluorescence and ER tracker staining in NC and circFAM13B knockdown cells. Scale bar, 10 µm. B. Quantification of RMB3-ER colocalization index by Pearson's correlation coefficient (PCC). **p*<0.05, ***p*<0.01 and ****p*<0.001.

Supplementary Figure 4: Transcriptome sequencing and cytokine chip sequencing of circFAM13B stable cells. A. KEGG functional enrichment analysis of DEGs. B. Transcriptome expression of cytokines in circFAM13B stable cell lines, the Y axis represents the FMPK value. C-E. KEGG (C) and GO (D, E) enrichment analysis of cytokine chip. **p*<0.05, ***p*<0.01 and ****p*<0.001.

Supplementary Figure 5: Splicing factor NOVA-1 promotes circFAM13B biogenesis. A. Knockdown of different splicing factors in HK1 cells. B. circFAM13B expression after transfection with various splicing factor shRNAs; C. The circFANM13B and linear FAM13B expression after NOVA-1 knockdown. The error bars represent standard deviations of three independent experiments. **p*<0.05, ***p*<0.01 and ****p*<0.001.

**Supplementary Figure 1**

**
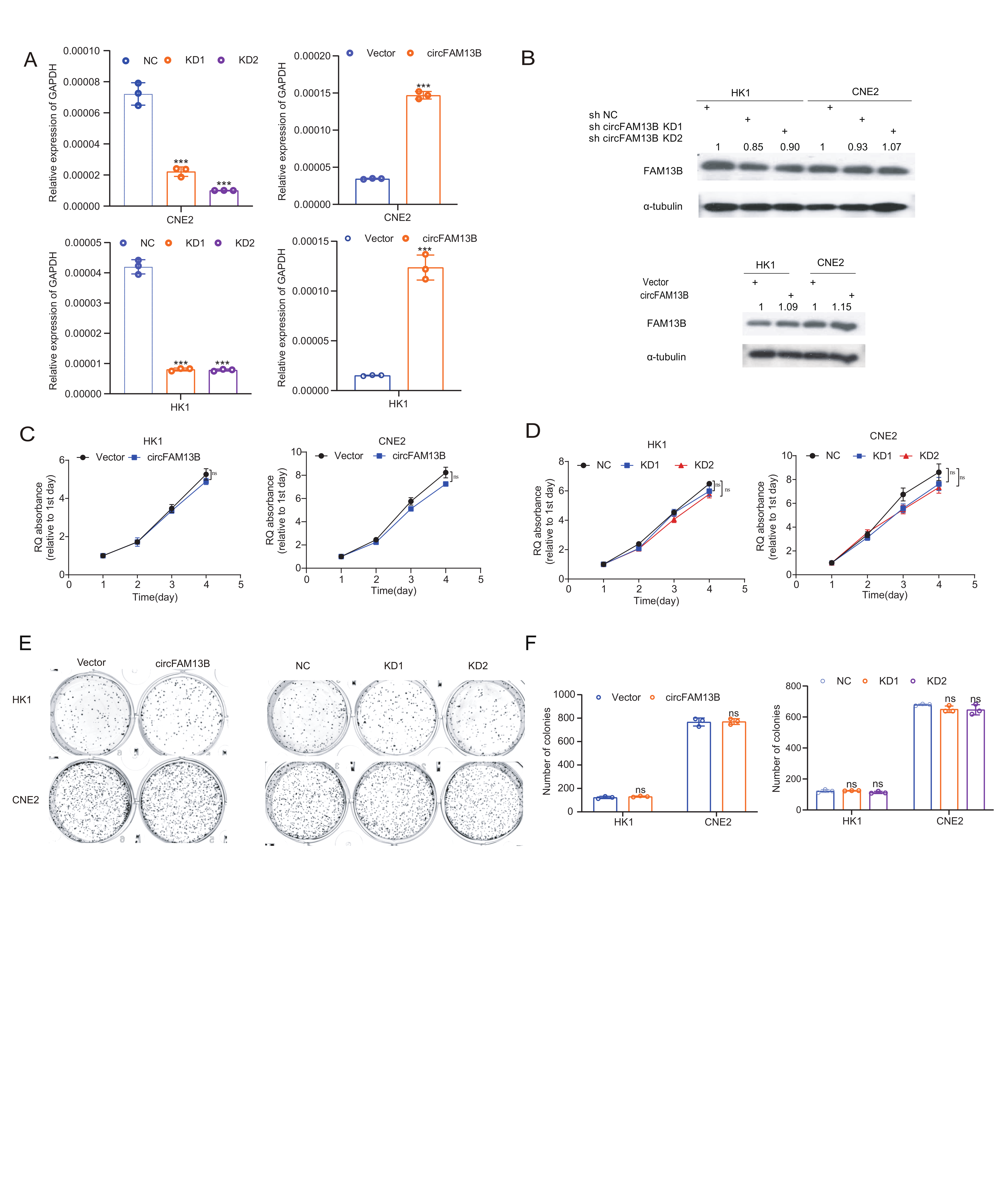
**

**Supplementary Figure 2**


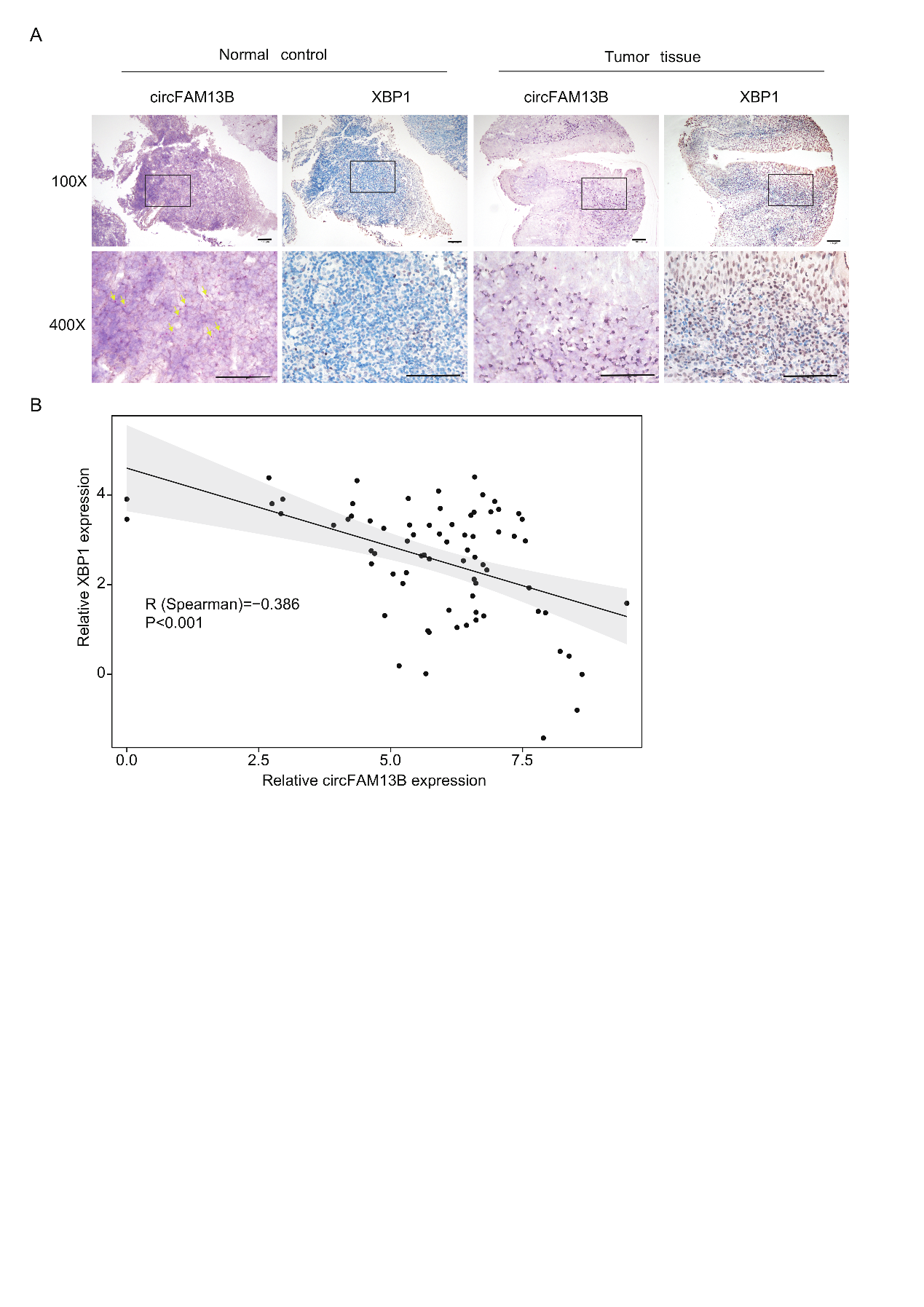


**Supplementary Figure 3**


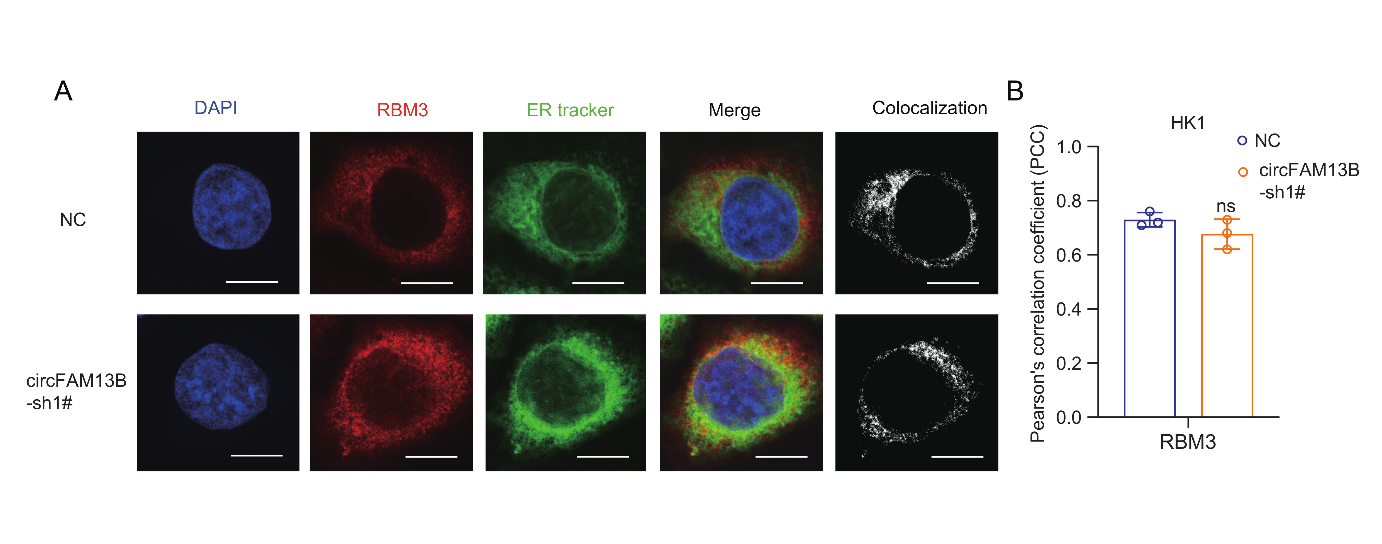


**Supplementary Figure 4**

**
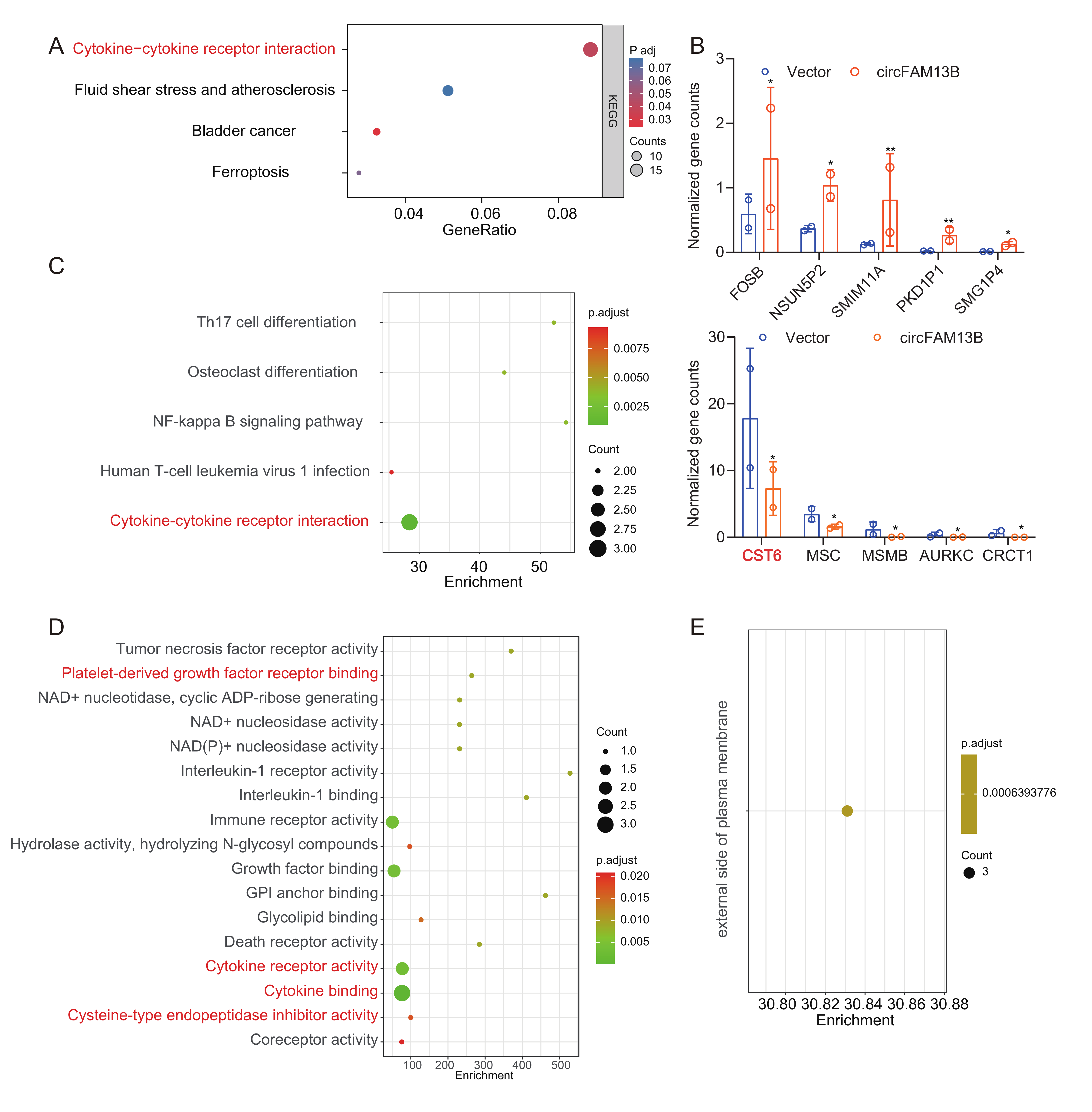
**

**Supplementary Figure 5**

**
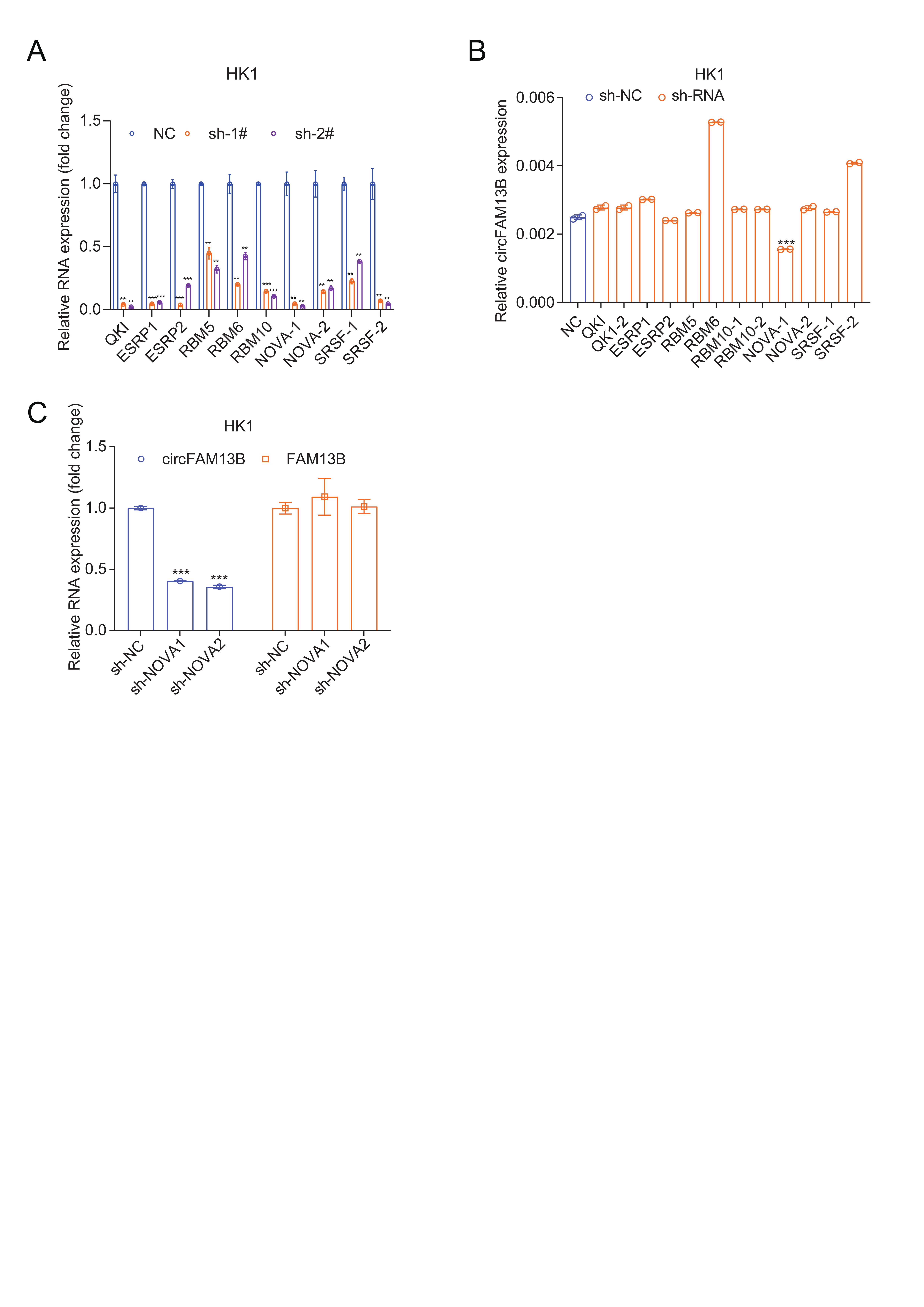
**

Supplementary Table 1: Summary of Serum Demographics of Patients with Nasopharyngeal Carcinoma

|  | **ALL** | **CST6 low** | **CST6 high** | ***P* value** |
| --- | --- | --- | --- | --- |
|  | ***N=168*** | ***N=78*** | ***N=90*** |  |
| Age, years |  |  |  | 0.301 |
| <45 | 75 (44.6%) | 31 (39.7%) | 44 (48.9%) |  |
| ≥45 | 93 (55.4%) | 47 (60.3%) | 46 (51.1%) |  |
| Sex |  |  |  | 0.501 |
| Male | 132 (78.6%) | 59 (75.6%) | 73 (81.1%) |  |
| Female | 36 (21.4%) | 19 (24.4%) | 17 (18.9%) |  |
| EBV DNA |  |  |  | 0.788 |
| <4000 copies/ml | 120 (71.4%) | 57 (73.1%) | 63 (70.0%) |  |
| ≥4000 copies/ml | 48 (28.6%) | 21 (26.9%) | 27 (30.0%) |  |
| Pathological type ^a^ |  |  |  | 0.249 |
| Keratinizing | 3 (1.79%) | 0 (0.00%) | 3 (3.33%) |  |
| Nonkeratinizing | 165 (98.2%) | 78 (100%) | 87 (96.7%) |  |
| T Stage |  |  |  | 0.577 |
| 1 | 2 (1.19%) | 0 (0.00%) | 2 (2.22%) |  |
| 2 | 14 (8.33%) | 5 (6.41%) | 9 (10.0%) |  |
| 3 | 100 (59.5%) | 49 (62.8%) | 51 (56.7%) |  |
| 4 | 52 (31.0%) | 24 (30.8%) | 28 (31.1%) |  |
| N Stage |  |  |  | **0.036** |
| 0 | 10 (5.95%) | 4 (5.13%) | 6 (6.67%) |  |
| 1 | 32 (19.0%) | 22 (28.2%) | 10 (11.1%) |  |
| 2 | 75 (44.6%) | 29 (37.2%) | 46 (51.1%) |  |
| 3 | 51 (30.4%) | 23 (29.5%) | 28 (31.1%) |  |
| Clinical Stage ^b^ |  |  |  | 0.812 |
| II | 3 (1.79%) | 2 (2.56%) | 1 (1.11%) |  |
| III | 79 (47.0%) | 37 (47.4%) | 42 (46.7%) |  |
| IVa | 86 (51.2%) | 39 (50.0%) | 47 (52.2%) |  |
| Smoking history |  |  |  | 0.623 |
| Never | 112 (66.7%) | 54 (69.2%) | 58 (64.4%) |  |
| Smoker | 56 (33.3%) | 24 (30.8%) | 32 (35.6%) |  |

^a^ Categorized according to the WHO Classification of Tumors.

^b^ Classified according to American Joint Committee on Cancer and Union for International Cancer Control version 8th TNM staging system.

All statistical tests were two-sided. *P-value was calculated with the Fisher’s exact test.

Supplementary Table 2: Summary of NPC Patient Tissue Microarray Demographics

|  | **ALL** | **circFAM13B low** | **circFAM13B high** | ***P* value** |
| --- | --- | --- | --- | --- |
|  | ***N=255*** | ***N=128*** | ***N=127*** |  |
| Age, years |  |  |  | 0.150 |
| <45 | 118 (46.3%) | 53 (41.4%) | 65 (51.2%) |  |
| ≥45 | 137 (53.7%) | 75 (58.6%) | 62 (48.8%) |  |
| Sex |  |  |  | 0.970 |
| Male | 188 (73.7%) | 95 (74.2%) | 93 (73.2%) |  |
| Female | 67 (26.3%) | 33 (25.8%) | 34 (26.8%) |  |
| EBV DNA |  |  |  | **0.013** |
| <4000 copies/ml | 195 (76.5%) | 89 (69.5%) | 106 (83.5%) |  |
| ≥4000 copies/ml | 60 (23.5%) | 39 (30.5%) | 21 (16.5%) |  |
| Pathological type ^a^ |  |  |  | 1.000 |
| Keratinizing | 2 (0.78%) | 1 (0.78%) | 1 (0.79%) |  |
| Nonkeratinizing | 253 (99.2%) | 127 (99.2%) | 126 (99.2%) |  |
| T Stage |  |  |  | **<0.001** |
| 1 | 1 (0.39%) | 0 (0.00%) | 1 (0.79%) |  |
| 2 | 48 (18.8%) | 8 (6.25%) | 40 (31.5%) |  |
| 3 | 172 (67.5%) | 97 (75.8%) | 75 (59.1%) |  |
| 4 | 34 (13.3%) | 23 (18.0%) | 11 (8.66%) |  |
| N Stage |  |  |  | **0.004** |
| 0 | 33 (12.9%) | 12 (9.38%) | 21 (16.5%) |  |
| 1 | 67 (26.3%) | 24 (18.8%) | 43 (33.9%) |  |
| 2 | 102 (40.0%) | 60 (46.9%) | 42 (33.1%) |  |
| 3 | 53 (20.8%) | 32 (25.0%) | 21 (16.5%) |  |
| Clinical Stage ^b^ |  |  |  | **<0.001** |
| 2 | 16 (6.27%) | 0 (0.00%) | 16 (12.6%) |  |
| 3 | 165 (64.7%) | 79 (61.7%) | 86 (67.7%) |  |
| 4 | 74 (29.0%) | 49 (38.3%) | 25 (19.7%) |  |
| Smoking history |  |  |  | 0.437 |
| Never | 178 (69.8%) | 86 (67.2%) | 92 (72.4%) |  |
| Smoker | 77 (30.2%) | 42 (32.8%) | 35 (27.6%) |  |

^a^ Categorized according to the WHO Classification of Tumors.

^b^ Classified according to American Joint Committee on Cancer and Union for International Cancer Control version 8th TNM staging system.

All statistical tests were two-sided. *P-value was calculated with the Fisher’s exact test.

**Supplementary Table 3: Results of univariate and multivariate analysis of clinical samples**

| **Characteristics** | **Total(N)** | **Univariate analysis** | |  | **Multivariate analysis** | |
| --- | --- | --- | --- | --- | --- | --- |
|  |  | **Hazard ratio (95% CI)** | ***P* value** |  | **Hazard ratio (95% CI)** | ***P* value** |
| circFAM13B expression | 255 |  |  |  | Reference |  |
| low | 128 | Reference |  |  | 0.450 (0.205 - 0.986) | **0.046** |
| high | 127 | 0.454 (0.221 - 0.932) | **0.031** |  |  |  |
| Sex | 255 |  |  |  | § |  |
| Male | 188 | Reference |  |  | § |  |
| Female | 67 | 0.646 (0.281 - 1.485) | 0.304 |  |  |  |
| Age, years | 255 |  |  |  | § |  |
| ≥45 | 137 | Reference |  |  | § |  |
| <45 | 118 | 0.652 (0.326 - 1.303) | 0.226 |  |  |  |
| T Stage ^a^ | 255 |  |  |  | Reference |  |
| 2 | 49 | Reference |  |  | 1.748 (0.579 - 5.273) | 0.322 |
| 4 | 34 | 2.641 (0.927 - 7.527) | 0.069 |  | 0.635 (0.233 - 1.728) | 0.374 |
| 3 | 172 | 0.866 (0.346 - 2.170) | 0.759 |  |  |  |
| N stage ^a^ | 255 |  |  |  | Reference |  |
| 0 | 202 | Reference |  |  | 1.438 (0.676 - 3.056) | 0.345 |
| 3 | 53 | 1.827 (0.861 - 3.874) | 0.116 |  |  |  |
| Pathological type ^b^ | 255 |  |  |  | § |  |
| Nonkeratinizing | 253 | Reference |  |  | § |  |
| Keratinizing | 2 | 4.220 (0.574 - 31.011) | 0.157 |  |  |  |
| EBV DNA | 255 |  |  |  | § |  |
| <4000 copies/ml | 195 | Reference |  |  | § |  |
| ≥4000 copies/ml | 60 | 0.644 (0.256 - 1.624) | 0.351 |  |  |  |
| Smoking | 255 |  |  |  | Reference |  |
| Never | 178 | Reference |  |  | 1.536 (0.770 - 3.067) | 0.223 |
| Smoker | 77 | 1.665 (0.840 - 3.301) | 0.144 |  | Reference |  |

^a^ Classified according to American Joint Committee on Cancer and Union for International Cancer Control version 8th TNM staging system.

^b^ Categorized according to the WHO Classification of Tumors.

P<.05(two-sided) stood for statistical significance. Besides, variables satisfying p<.2 upon univariate analysis were chosen to be potential variables for multivariate regression.

**Supplementary Table 4. List of primers.**

| **Primer-id** | **Sequence** |
| --- | --- |
| circFAM13B pF | 5’- AATGAAGAAAATACCCAGCACCC -3’ |
| circFAM13B pR | 5’- ACACACCACACTTTGCTGTTGTAAA -3’ |
| FAM13B pF | 5’- TTCAGAGCAGCATAGATCAT -3’ |
| FAM13B pF | 5’- CATCAGCAATATCAATCTGG -3’ |
| Divergent-GAPDH pF | 5’- CCAATACGACCAAATCCGTT-3’ |
| Divergent-GAPDH pR | 5’- CGAGATCCCTCCAAAATCAA-3’ |
| GAPDH pF | 5’- TGGGCAGCCGTTAGGAAAG-3’ |
| GAPDH pR | 5’- GACTCCACGACGTACTCAGC-3’ |
| XBP1 pF | 5’- GGAACAGCAAGTGGTAGA -3’ |
| XBP1 pR | 5’- CTGGAGGGGTGACAACTG -3’ |
| uXBP1 pF | 5’- CAGACTACGTGCGCCTCTGC -3’ |
| uXBP1 pF | 5’- CTTCTGGGTAGACCTCTGGG -3’ |
| sXBP1 pF | 5’- TCTGCTGAGTCCGCAGCAGG-3’ |
| sXBP1 pR | 5’- CTCTAAGACTAGAGGCTTGG-3’ |
| IRE1α pF | 5’- TTCCCAGAGCCGTTCCTTTC-3’ |
| IRE1α PR | 5’- GGCTGCAAAAGTGGAACTGG-3’ |
| RBM3 pF | 5’- GGGCAGAGATGCACTGAACT-3’ |
| RBM3 PR | 5’- AGGGAATAGGCAGCTCAGGA-3’ |
| CST6 pF | 5’- TACTTCCGAGACACGCACAT -3’ |
| CST6 pR | 5’- CTCCCCATCTCCATCGTCAG -3’ |
| CST6-1 pF | 5’- CTGGCAATTATTGGAGTCTT -3’ |
| CST6-1 pR | 5’- CGTGGATACAGTGAGCAT -3’ |
| CST6-2 pF | 5’- AAGCCATGCCGTCAGTAA-3’ |
| CST6-2 pR | 5’- GAGCCCACAGGACACAAA-3’ |
| CST6-3 pF | 5’- CCACAGATTTGACTTAGACTG-3’ |
| CST6-3 pR | 5’- GTTTCCACTTTACAGAGAAGG-3’ |
| CST6-4 pF | 5’- TCTGTCACCCAGGCTAGA -3’ |
| CST6-4 pR | 5’- GTGGCACACACCTGAATT -3’ |
| CST6-5 pF | 5’- CAGGCTGGTCTTGAACTC-3’ |
| CST6-5 pR | 5’- AGGGCTCACACTTGTAATC-3’ |
| CST6-6 pF | 5’- TCACGATTCACTGACTTGA-3’ |
| CST6-6 pR | 5’- GCTTGTAATTCCAGCACTT-3’ |
| CST6-7 pF | 5’- CTCCAGGAAGGTTGTCAG-3’ |
| CST6-7 pR | 5’- TGTTGTGAGGATTCAGTGA-3’ |
| CST6-8 pF | 5’- AGTGACTTCTCCGAGGTTA-3’ |
| CST6-8 pR | 5’- GGCTGTCCAGAATGAACTA-3’ |

CST6 1-8 is the sequence for verification of CST6 promoter truncation in CHIP experiment.

**Supplementary Table 5. List of antibodies.**

| **Antibody** | **Company** | **Catalog Number** |
| --- | --- | --- |
| Anti-mouse IgG, HRP-linked antibody | Cell Signaling Technology | 7076 |
| Anti-rabbit IgG, HRP-linked Antibody | Cell Signaling Technology | 7074 |
| ACTIN | Cell Signaling Technology | 3700 |
| Tublin | Cell Signaling Technology | 2148 |
| Anti-XBP1 | Abcam | Ab220783 |
| Anti-P-IRE1a | Abcam | Ab124945 |
| Anti-IRE1a | Cell Signaling Technology | 3294 |
| Anti-ATF6 | Abcam | Ab174756 |
| Anti-P-EIF2a | Abcam | Ab32157 |
| Anti-EIF2a | Abcam | Ab5369 |
| Anti-FAM13B | Abmart | PC5757 |
| Anti-RBM3 | Abcam | Ab134946 |
| Human Cystatin E/M Antibody | Bio-Techne | MAB1286 |
| Goat anti-Rabbit IgG (H+L) Cross-Adsorbed Secondary Antibody, Alexa Fluor™ 488 | Invitrogen | A11004 |
| Goat anti-Mouse IgG (H+L) Cross-Adsorbed Secondary Antibody, Alexa Fluor™ 568 | Invitrogen | A11008 |
| GAPDH | Abclonal | AC002 |
